# Linkage Engineering Boosts Battery Performance of Two‐Electron Phenothiazine‐Based Polyamide Cathodes

**DOI:** 10.1002/advs.77960

**Published:** 2026-09-27

**Authors:** Shujuan Cao, Chunping Ren, Jing Qin, Xiujuan Wang, Xiaoming He

**Affiliations:** ^1^ Key Laboratory of Applied Surface and Colloid Chemistry (Ministry of Education) School of Chemistry and Chemical Engineering Shaanxi Normal University Xi'an People's Republic of China

**Keywords:** linkage engineering, phenothiazine, polyamides, p‐type electrode, two electron

## Abstract

Organic cathode materials based on redox‐active p‐type phenothiazine have attracted considerable interest for lithium‐organic batteries (LOBs) due to their high operating voltage and tunable chemical structures. Nevertheless, their practical application is often limited by low capacity resulting from single‐electron transfer per molecule, as well as poor cycling stability caused by solubility issues. In this work, we report three phenothiazine‐based polyamides and demonstrate a straightforward linker engineering strategy to synergistically overcome these challenges. The linker motif serves not only as a structural bridge to suppress solubility, but also as a functional design element that governs optoelectronic properties and electron/ion transport kinetics. Among the series, the benzene‐bridged polymer **MPT‐AB** exhibits the most balanced optoelectronic performance and transport kinetics, resulting in the best battery performance. When employed as a cathode in LOBs, **MPT‐AB** exhibits two reversible redox processes, a high average voltage of 3.7 V, and a reversible capacity of 150 mAh g^−1^ at 0.2 A g^−1^. It also demonstrates excellent rate capability and outstanding cycling stability, achieving 90 % and 72% capacity retention after 2000 and 5000 cycles at 5 A g^−1^. The potential application has also been demonstrated with satisfactory performance in an **MPT‐AB**//graphite full battery.

## Graphical abstract

Three phenothiazine‐based polyamides featuring gradually strengthened electron‐withdrawing linkers are developed as high‐voltage cathodes for lithium‐organic batteries. The linker motif serves not only as a structural bridge to suppress solubility, but also as a functional design element that governs optoelectronic properties and electron/ion transport kinetics.

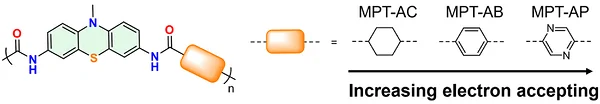

## 1 Introduction

The global effort to reduce carbon emissions and decrease reliance on critical metal resources used in secondary battery manufacturing has attracted significant attention. In response to these challenges, battery technology innovation is increasingly moving beyond conventional lithium‐ion batteries (LIBs) toward novel electrode materials and alternative systems. In this context, redox‐active organic electrode materials (OEMs) offer distinct advantages, including the potential for sustainable sourcing from natural products and a low carbon footprint [1, 2, 3, 4]. Based on their charge‐storage mechanisms, OEMs are generally classified into three categories: n‐type, p‐type, and bipolar type. Widely reported compounds such as carbonyls [5, 6, 7, 8], imines [9, 10, 11], and viologens [12, 13, 14, 15] fall under the n‐type category; however, they typically exhibit relatively low redox potentials (below 3.0 V vs. Li/Li^+^), which are significantly lower than those of conventional inorganic cathodes (3.4–4.1 V vs. Li/Li^+^). In contrast, p‐type OEMs, including triphenylamine derivatives, organosulfides, and phenazine, operate through electron loss and present excellent opportunities for achieving high voltages (exceeding 3.5 V) in rechargeable batteries [16, 17, 18, 19, 20, 21, 22, 23, 24, 25, 26]. Nevertheless, the main challenge for p‐type OEMs lies in their low specific capacity, and to date, only a limited number of such materials have been thoroughly investigated.

**SCHEME 1:**
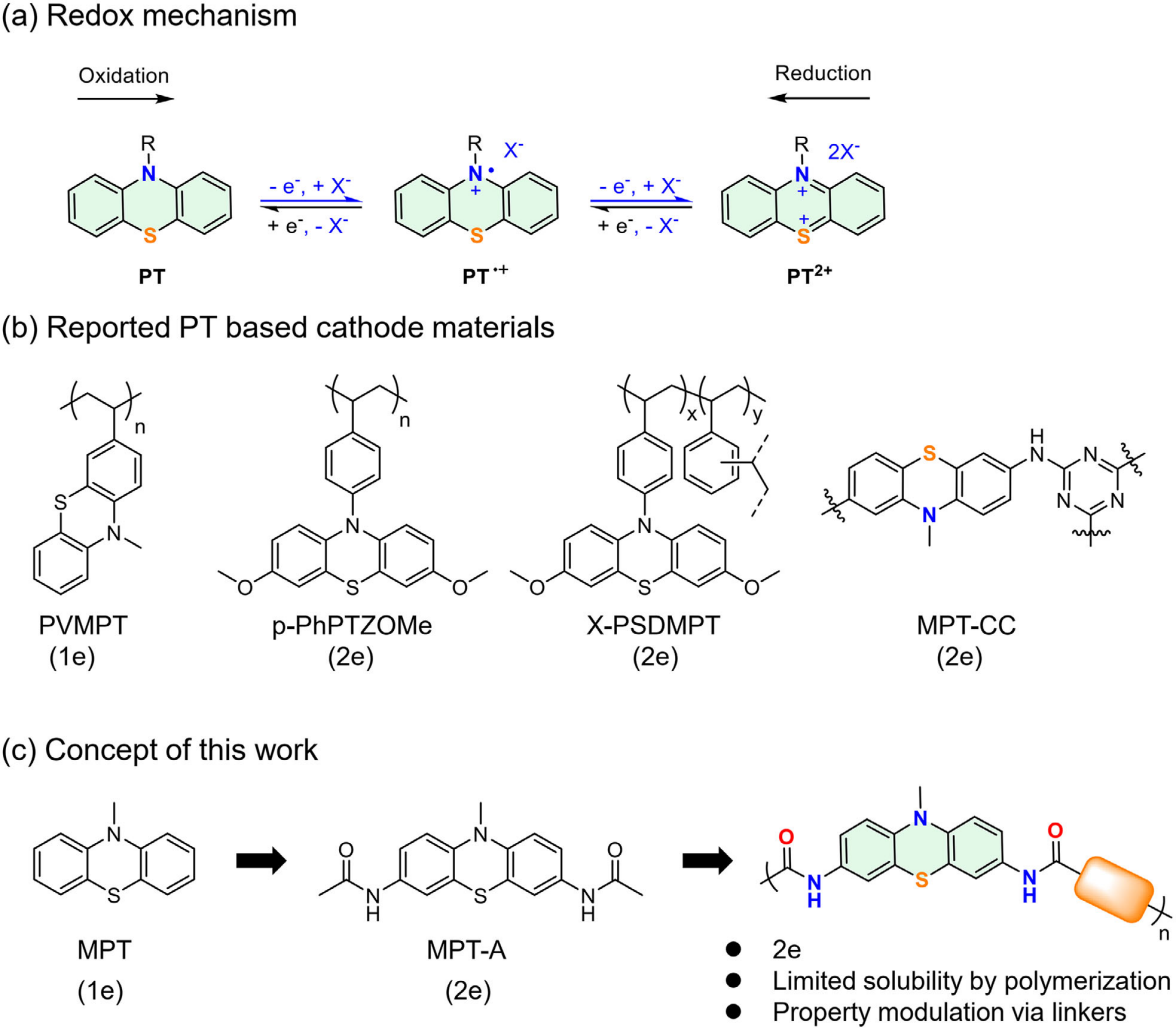
(a) Two‐step redox process of phenothiazine (PT). (b) Reported PT‐based cathode materials. (c) Concept of this work.

Phenothiazines (PT) represent a promising and scalable platform for constructing p‐type OEMs, owing to their high electrochemical reversibility and versatile chemical tunability [27, 28, 29, 30, 31, 32, 33]. PT compounds can undergo a two‐step oxidation process, first forming a radical cation (PT^•+^) and then progressing to a dication (PT^2+^), as shown in Scheme 1a. However, the oxidation to the dication state occurs at a relatively high redox potential and is prone to rapid decomposition, which hinders its practical utilization. As a result, most reported PT‐based organic cathodes primarily rely on the PT/PT^•+^ redox couple, limiting their capacity to only one redox event per molecule. A representative phenothiazine‐based one‐redox electrode, PVMPT (Scheme 1b), reported by Esser, delivers a high discharge potential of 3.5 V (vs. Li/Li^+^), yet exhibits a limited specific capacity of only 56 mAh g^−1^ [28]. Therefore, achieving reversible two‐electron transfer in phenothiazine‐based electrodes is highly desirable, as it could double the delivered capacity and enable high‐performance lithium‐organic batteries (LOBs).

So far, reported phenothiazine systems with reversible two‐electron oxidation properties remain highly limited. In 2024, the groups of Zhao and Esser reported linear and cross‐linked poly(N‐styryl‐3,7‐dimethoxyphenothiazine) polymers p‐PhPTZOMe and X‐PSDMPT (Scheme 1b), which showed excellent performance in LOBs [34, 35]. Both works demonstrated that electron‐donating methoxy substituents on the phenothiazine backbone stabilize the dicationic state through extended charge delocalization. Although the polymer design can significantly suppress solubility in the electrolyte, the preparation involves complicated and multi‐step synthetic procedures.

3,7‐diamino‐N‐methyl phenothiazine (MPT‐NH_2_) has garnered our interest due to its promising electrochemical properties. The introduction of electron‐donating amine substituents to the phenothiazine skeleton can increase the electron density and shift the second oxidation process into a stable range. Moreover, the presence of reactive amine groups enables diverse chemical modifications, making this derivative highly suitable for constructing redox‐active polymers. We recently developed a MPT‐CC polymer (Scheme 1b), prepared via a simple one‐step dehydrochlorination reaction of cyanuric chloride with MPT‐NH_2_, as a promising two‐electron organic cathode material [36].

A previous work by Guilmin et al. demonstrated that modifying the NH_2_ functionality of MPT‐NH_2_ via amidation preserves two reversible redox processes [37]. This finding motivates our current investigation into phenothiazine‐based polyamides as two‐electron cathode materials (Scheme 1c). In this work, we report the facile synthesis of three phenothiazine‐based polyamides (**MPT‐AC**, **MPT‐AB,** and **MPT‐AP**), and demonstrate a straightforward linkage engineering strategy to improve LOB performance. These polymers feature a similar topology with phenothiazine as the redox center, while incorporating electronically distinct linkages (cyclohexane, benzene, and pyrazine) with increasing electron‐withdrawing character. The linkage motif serves a dual purpose: it acts not only as a structural bridge to limit solubility but also as a functional design element that regulates optoelectronic properties and electron/ion transport kinetics. Within the series, the benzene‐linked polymer **MPT‐AB** achieves the best battery performance, attributed to its well‐balanced optoelectronic properties and favorable electron/ion transport kinetics. When used as a cathode in LOBs, **MPT‐AB** delivers a high reversible capacity of 150 mAh g^−1^ at 0.2 A g^−1^, outstanding rate capability, and exceptional cycling stability. This study underscores linkage engineering as an effective approach for tuning the structural and electronic characteristics of organic polymers, paving the way for advanced battery material design.

## 2 Results and Discussion

### 2.1 Design, Synthesis, and Characterization

**FIGURE 1:**
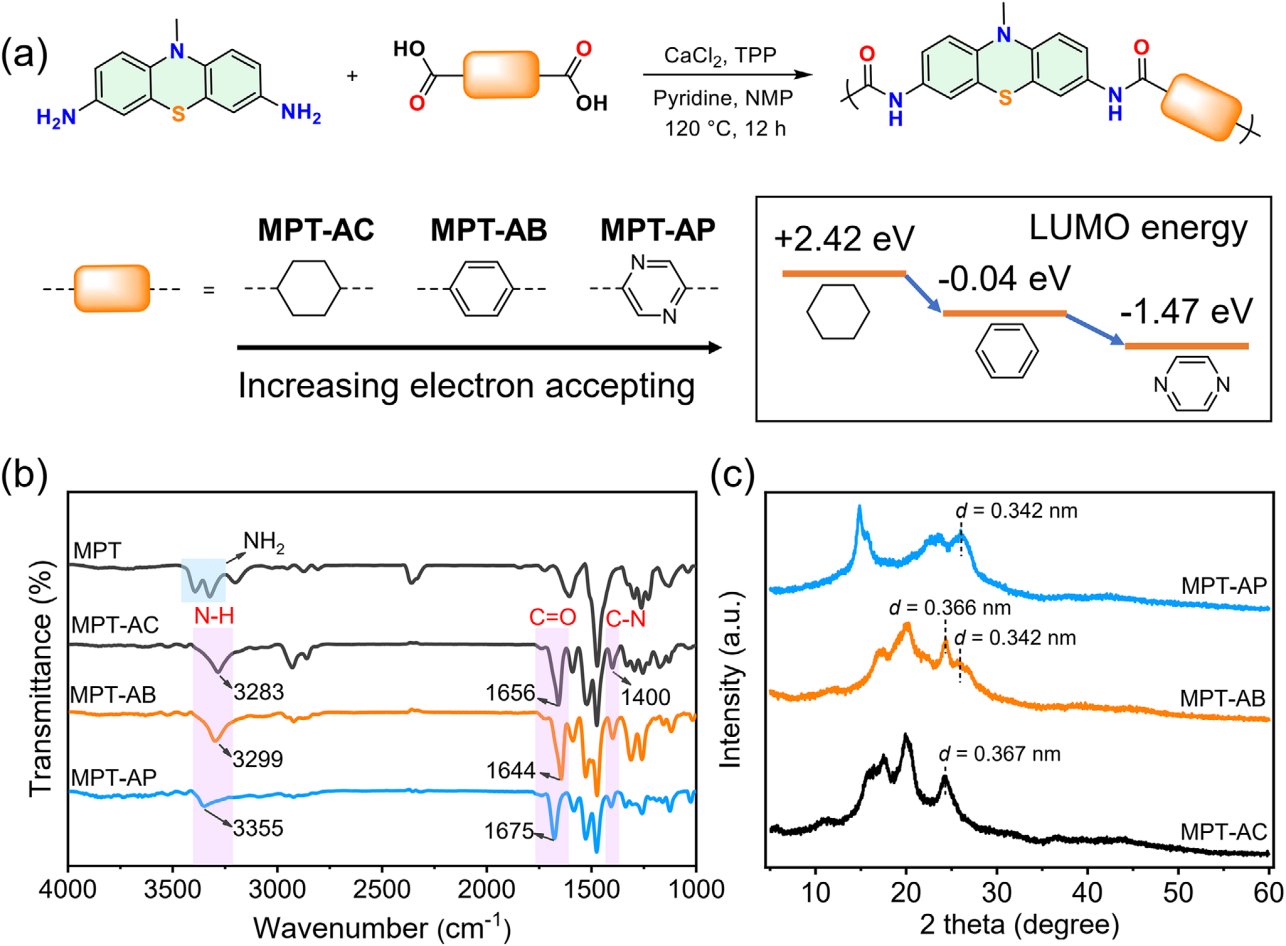
(a) Design and synthesis, (b) FT‐IR spectra, (c) XRD spectra of phenothiazine‐based polyamides MPT‐AC, MPT‐AB, and MPT‐AP.

Three phenothiazine‐based polyamides (**MPT‐AC**, **MPT‐AB**, and **MPT‐AP**, Figure 1a) were rationally designed and facilely prepared in high yields (>95%) via a phosphorylation polyamidation approach [38, 39, 40], using MPT‐NH_2_ and various diacids. These diacids, featuring distinct linkers (cyclohexane, benzene, and pyrazine), were deliberately chosen for their progressively increasing electron‐withdrawing characteristics, as confirmed by their gradually decreasing LUMO energies. The solubility of the three polymers is poor in many common organic solvents, which can be attributed to the strong interchain interactions arising from *π–π* stacking and hydrogen bonding (vide infra). Consequently, the resulting polymers, which share similar topologies, provide an ideal platform to investigate the effects of linker engineering on governing electrochemistry and electron/ion transport kinetics.

The successful construction of three polyamides was confirmed by multiple characterization methods. The Fourier transform infrared (FT‐IR) spectra of the three polymers and MPT‐NH_2_ were compared to further elucidate the functional group information (Figure 1b). Three polymers exhibited IR bands of the amide group at around 3283–3355 cm^−1^ for N‐H stretching and 1644–1675 cm^−1^ for amide C = O. Moreover, the absorption peaks of the NH_2_ group at 3391 and 3320 cm^−1^ for MPT‐NH_2_, disappeared in the polymer spectra. These results confirm the complete conversion of the amino group into the amide group.

The x‐ray diffraction (XRD) patterns of the three polymers exhibit several peaks at 2θ angles in the range of 10–30°, indicating their crystalline nature (Figure 1c). Notably, tuning the linkers from cyclohexane to pyrazine leads to closer *π–π* stacking with shorter d‐space, which can be attributed to the enhanced electron‐withdrawing property that enables stronger interactions with phenothiazine between polymer chains. Thermogravimetric analysis (TGA) indicated that **MPT‐AC** and **MPT‐AB** have better thermal stability than **MPT‐AP**, with decomposition temperatures (*T*_d_, 5% weight loss) of 304°C for the former two and 238°C for the latter under an N_2_ atmosphere (Figure S1).

### 2.2 Model Compound Study

**FIGURE 2:**
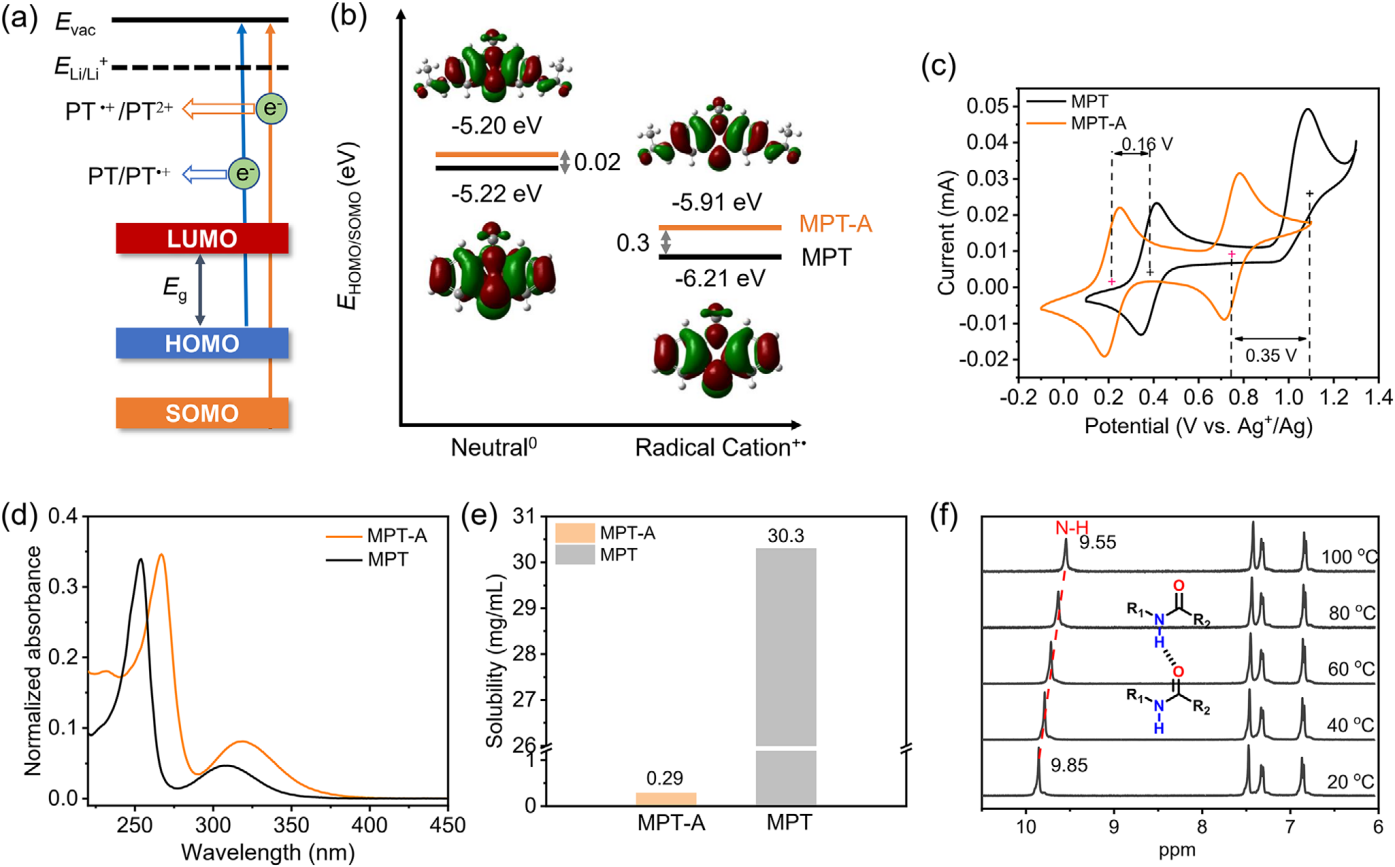
(a) Relation between frontier orbitals and two redox reactions of PT. (b) DFT‐calculated HOMO and SOMO energies of MPT‐A (yellow) and MPT (black). (c) CV spectra, (d) UV–vis spectra, and (e) solubility of MPT‐A and MPT in CH_3_CN. (f) ^1^H NMR spectra of MPT‐A at variable temperatures of 20–100 °C. Inset shows the hydrogen bond between amides.

In p‐type redox reactions, electron loss takes place from the highest‐energy orbital. The redox potential is theoretically determined by the energy levels of the highest occupied molecular orbital (HOMO) for the first redox reaction, and by the singly occupied molecular orbital (SOMO) for the second (Figure 2a) [16, 34]. For most phenothiazine derivatives, the first oxidation (PT^0^/ PT^•+^) is reversible, whereas the reversibility of the second oxidation (PT^•+^/PT^2+^) strongly depends on the substituents.

Figure 2b illustrates the HOMO and SOMO energy levels of N‐methyl phenothiazine (MPT) and its acetamide‐substituted derivative (**MPT‐A**), serving as a model compound to investigate the effect of amide substitution on the electronic properties of the phenothiazine core. Compared to **MPT**, the introduction of two amide groups elevates the *E*_HOMO_ and *E*_SOMO_ of **MPT‐A** by 0.02 and 0.3 eV, suggesting a lowering of the oxidation potential, especially for the second oxidation. Molecular orbital analysis of **MPT‐A** reveals that the amide substituents contribute partial electron density to the SOMO orbital, thereby raising the *E*_SOMO_. This suggests that **MPT‐A** is more likely to form stable PT^2+^ species, enabling reversible two‐step oxidation.

To validate the two‐electron process, **MPT‐A** was synthesized, and detailed preparation and characterization (Figures S2–S4) are included in the Supporting Information. The electrochemical properties of **MPT** and **MPT‐A** were comparatively investigated in CH_3_CN solution (Figure 2c). **MPT‐A** exhibits two reversible oxidations, whereas the second oxidation of **MPT** is not reversible. Relative to **MPT**, the two redox potentials of **MPT‐A** (0.21 and 0.75 V vs. Ag^+^/Ag) are negatively shifted by 0.16 and 0.35 V, respectively. Additionally, the UV–vis spectrum of **MPT‐A** shows a distinct redshift, suggesting extended conjugation (Figure 2d). These results are consistent with DFT calculations, indicating that the amide group acts as an electron donor that facilitates both oxidation processes of phenothiazine.

Notably, the solubility of **MPT‐A** in acetonitrile is about 100‐fold lower than that of **MPT** (**MPT‐A**: 0.29 mg/mL, **MPT**: 30.3 mg/mL, Figure 2e). This marked reduction in solubility can be attributed to intermolecular hydrogen bonding interactions. To confirm this, a temperature‐dependent ^1^H NMR experiment was carried out (Figure 2f). Increasing the temperature from 20°C to 100°C led to the obvious NH upfield shifts of the amide for **MPT‐A** by 0.2–0.3 ppm, indicating the disruption of the intermolecular hydrogen bonds [41, 42]. These results partially support the presence of hydrogen bonds in three polymers, partially accounting for their insolubility characteristics. Overall, these results substantiate our molecular design strategy and suggest enhanced potential for maintaining long‐term cycling stability.

### 2.3 LOB Performance

To evaluate the performance of three designed polymers as cathodes for LOBs, we fabricated half‐cell batteries with a Li anode. The working electrodes consist of 6:3:1 (mass ratio) active material, Ketjen black (ECP‐600J) as the conductive additive, and polytetrafluoroethylene (PTFE) as the binder, and the electrolyte is 1 m LiPF_6_ in EC:DMC:EMC (1:1:1, v/v).

**FIGURE 3:**
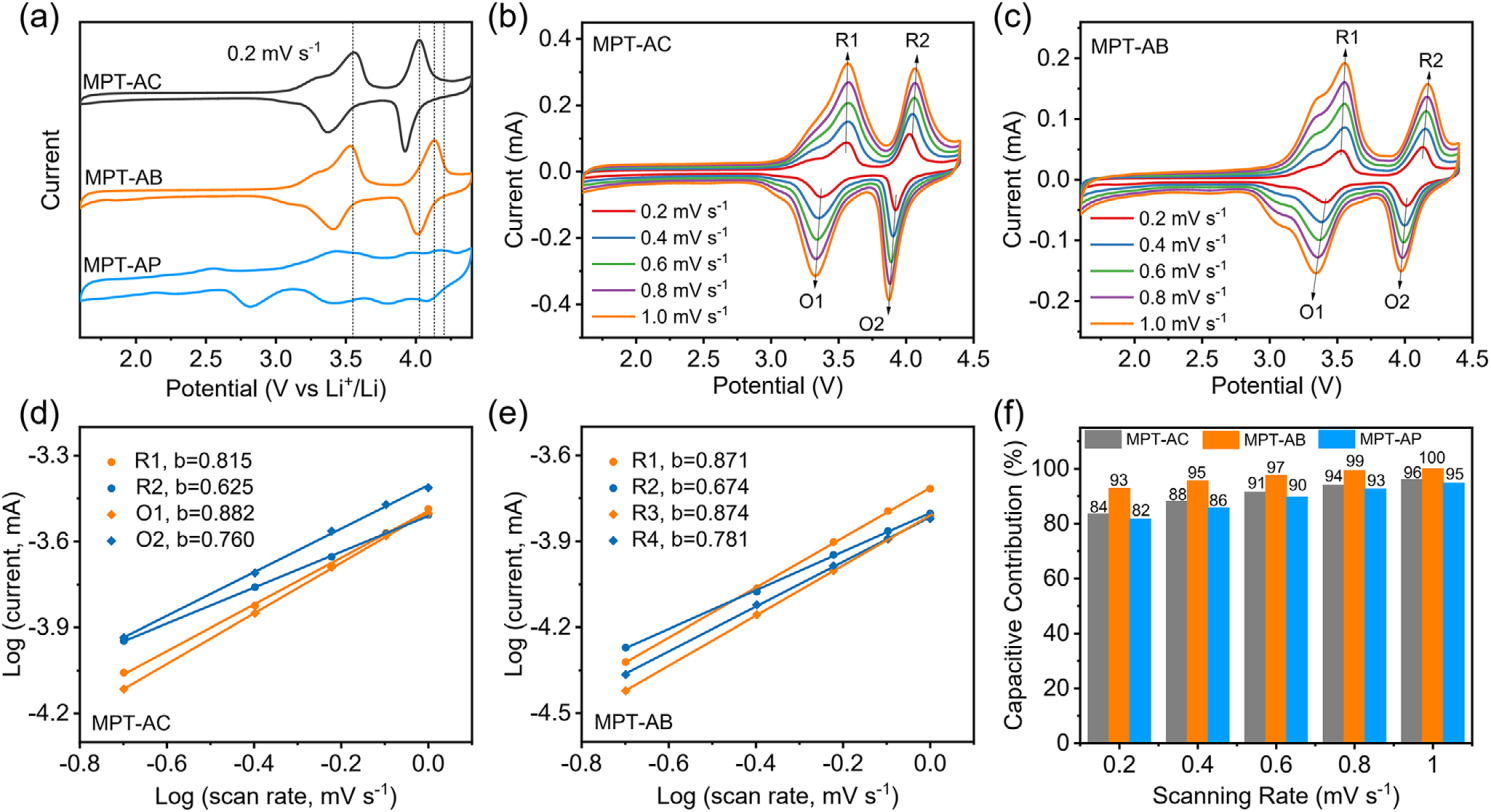
(a) Comparison of CV curves of MPT‐AC, MPT‐AB, and MPT‐AP at 0.2 mV s^−1^. (b, c) CV curves of (b) MPT‐AC and (c) MPT‐AB at different scan rates of 0.2–1.0 mV s^−1^. (d, e) Plot of log(i) vs. log(v) of multiple redox peaks for (d) MPT‐AC and (e) MPT‐AB. (f) Capacitive contributions (in percentage) of MPT‐AC, MPT‐AB, and MPT‐AP at different scan rates.

The redox potential and electrochemical activity of three cells were comparatively evaluated by cyclic voltammetry (CV) within the voltage range of 1.6–4.4 V vs. Li/Li^+^ at 0.2 mV s^−1^ (Figure 3a). The **MPT‐AC** electrode exhibits reversible double redox reactions at 3.46 and 3.96 V (vs. Li/Li^+^). With enhanced electron‐withdrawing linkers, **MPT‐AB** and **MPT‐AP** have higher redox potentials, particularly for the second one (PT^•+^/PT^2+^). This behavior is attributed to the reduced electron density on the phenothiazine unit, caused by intra‐ or intermolecular charge transfer to the electron‐withdrawing linkers. Notably, **MPT‐AB** still retains two reversible redox pairs, whereas the strongest electron‐withdrawing pyrazine linker raises the second oxidation potential to the highest value, making the PT^•+^/PT^2+^ process quasi‐reversible. Additionally, an extra redox pair at ca. 2.8 V, which is ascribed to the redox reaction of the dicarbonyl pyrazine moiety, is observed for **MPT‐AP**. These results demonstrate that linker engineering can effectively modulate the electrochemical behavior of phenothiazine‐based polyamides. Importantly, the two redox reactions of **MPT‐AC** and **MPT‐AB** remain within a stable operating window and can be fully utilized.

Their CV profiles are well retained at elevated scan rates, albeit with modest polarization, indicating good electrochemical stability (Figure 3b,c and Figure S5). The dependence of peak current on scan rate (*i* = *a*v^b^) was used to evaluate charge storage kinetics [43]. As shown in Figure 3d,e, linear fitting of log(*v*) vs. log(*i*) yields slopes of 0.62–0.88 for MPT‐AC and 0.67–0.87 for **MPT‐AB**, indicating synergistic contributions from diffusion‐controlled and capacitive‐controlled redox processes. More quantitatively, the capacitive contributions of **MPT‐AC** and **MPT‐AB** are determined to be 84% and 93% at 0.2 mV s^−1^, respectively, and these ratios increase to 96% and 100% at a higher scan rate of 1.0 mV s^−1^ (Figure 6f and Figures S6–S8). These results demonstrate a superior redox response arising from the capacitive‐controlled contribution, and indicate fast redox kinetics and efficient ion transport, which are beneficial for battery performance.

**FIGURE 4:**
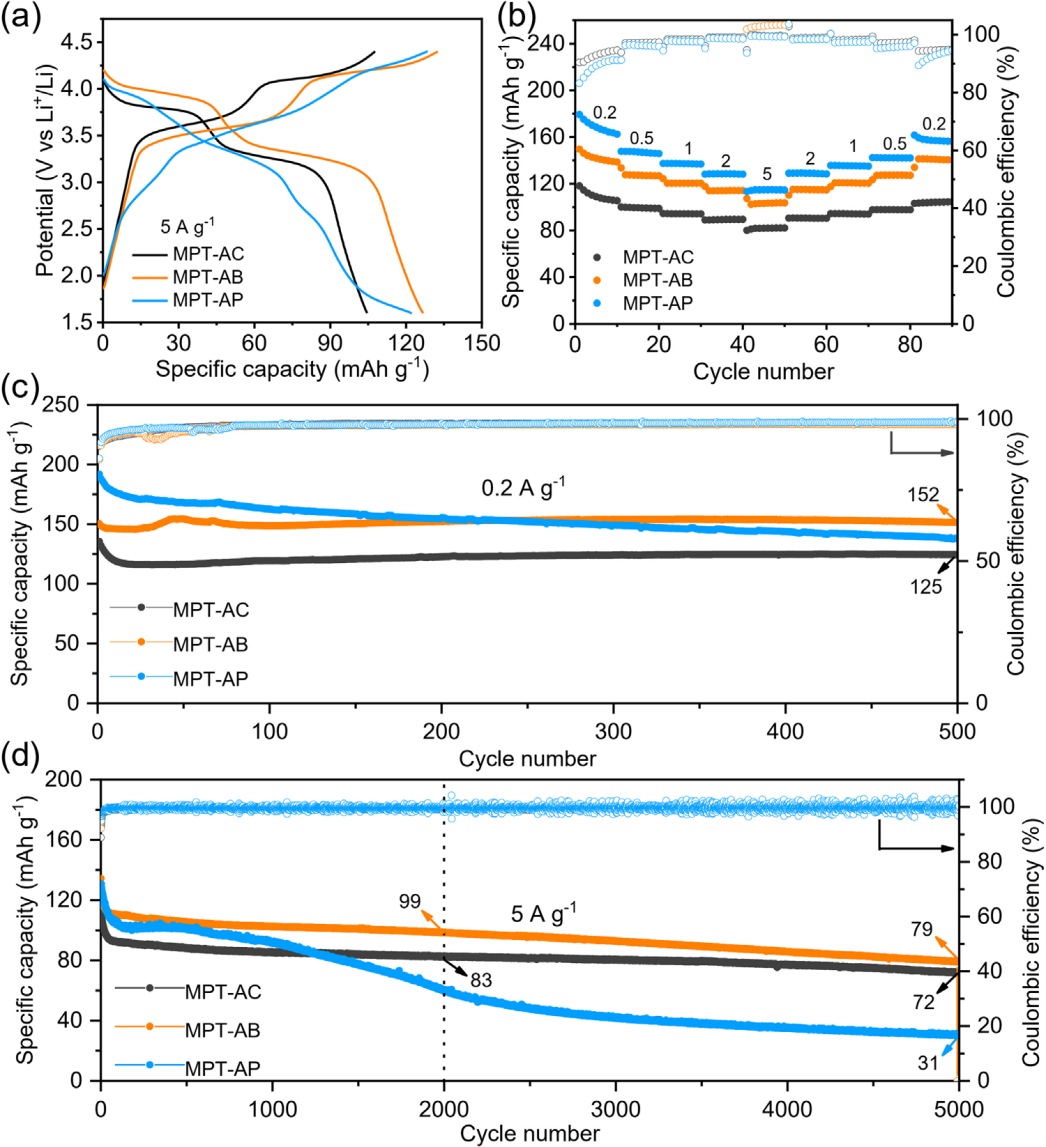
LOB performance of MPT‐AC, MPT‐AB, and MPT‐AP electrodes in a battery configuration (6:3:1 active material/Ketjin black/PTFE). (a) Charge–discharge curves at 5 A g^−1^. (b) Rate performance at the current from 0.2 to 5 A g^−1^. (c, d) Cycling performance and coulombic efficiency at (c) 0.2 A g^−1^ and (d) 5 A g^−1^.

Based on the understanding of their electrochemistry, the battery performance of the three polymers was further evaluated. Figure 4a shows the galvanostatic charge–discharge (GCD) curves of the three electrodes at 5 A g**^−^** ^1^. In agreement with the CV results, **MPT‐AC** and **MPT‐AB** exhibit two distinct plateaus, whereas **MPT‐AP** displays a sloped discharge curve with multiple short plateaus. The average voltages for **MPT‐AC** and **MPT‐AB** are 3.6 and 3.7 V, respectively. The slightly higher voltage of the **MPT‐AB** is attributed to the stronger electron‐accepting character of the benzene linkers, which is consistent with the CV results.

The rate performance of the electrodes was evaluated at various current densities ranging from 0.2 to 5.0 A g^−1^. As shown in Figure 4b, the discharge capacity follows the order of **MPT‐AP** > **MPT‐AB** > **MPT‐AC**. Specifically, at a current density of 0.2 A g^−1^, the **MPT‐AP**, **MPT‐AB,** and **MPT‐AC** electrodes deliver specific capacities of 179, 150, and 118 mAh g^−1^, respectively. Based on our previous work [36], the capacity contribution of Ketjen black is about 20 mAh g^−1^ at 0.2 A g^−1^ in the voltage range of 1.6–4.4 V. When the current density is increased from 0.2 to 5.0 A g^−1^, all three electrodes retain about 64%–69% of their initial capacity, demonstrating excellent rate capability (Table S1). Moreover, after high‐rate cycling, their capacities are almost fully recovered upon returning to 0.2 A g^−1^, indicating good structural reversibility. The highest capacity of **MPT‐AP** can be attributed to the additional contribution from the dicarbonyl pyrazine moiety. Compared with **MPT‐AC**, the higher capacity of **MPT‐AB** suggests more efficient utilization of the redox centers, likely due to its extended conjugation, which enhances electronic conductivity (vide infra).

Furthermore, the long‐term cycling stability of the three materials was systematically investigated. **MPT‐AB** and **MPT‐AC** exhibited superior cycling stability, in stark contrast to the rapid capacity decay observed for **MPT‐AP**. Specifically, **MPT‐AB** and **MPT‐AC** maintained reversible capacities of 152 and 125 mAh g^−1^, respectively, after 500 cycles at 0.2 A g^−1^, with capacity retentions close to 100%. The coulombic efficiencies of both materials remain close to 100%, confirming highly reversible redox stability. Throughout the cycling process, **MPT‐AB** consistently delivered a higher capacity than **MPT‐AC**, which is consistent with its superior rate performance and further indicates faster electronic and ionic conductivity. Moreover, even at a high current density of 5 A g^−1^, the **MPT‐AB** electrode achieved a reversible capacity of 99 mAh g^−1^ (90% retention) after 2000 cycles and 79 mAh g^−1^ (72% retention) after 5000 cycles. In contrast, the **MPT‐AP** electrode exhibited rapid capacity decay, retaining only a low capacity of 31 mAh g^−1^ after 5000 cycles at 5 A g^−1^. This poor stability can be partially attributed to the solubility of **MPT‐AP** in the electrolyte. As shown in Figure S9, the electrolyte solution of **MPT‐AP** appears yellow, while those of the other two polymers remain colorless. Additionally, its high redox potential may lead to decomposition of materials during cycling, leading to poor cycling stability.

In addition, the battery performance of the model compound **MPT‐A** was also investigated. As expected, **MPT‐A** exhibits two distinct voltage plateaus, similar to those observed for the polymers **MPT‐AC** and **MPT‐AB** (Figure S10a). Notably, although **MPT‐A** demonstrates relatively stable cycling performance over 1500 cycles at 5 A g^−^^1^, it delivers a low specific capacity of only 62 mAh g^−1^ (Figure S10b). These results suggest that the abundant amide groups in the polymer backbone can provide sufficient anion transport, facilitating rapid ion migration within the electrode material and thereby contributing to its high capacity.

### 2.4 Charge‐Storage Kinetics

**FIGURE 5:**
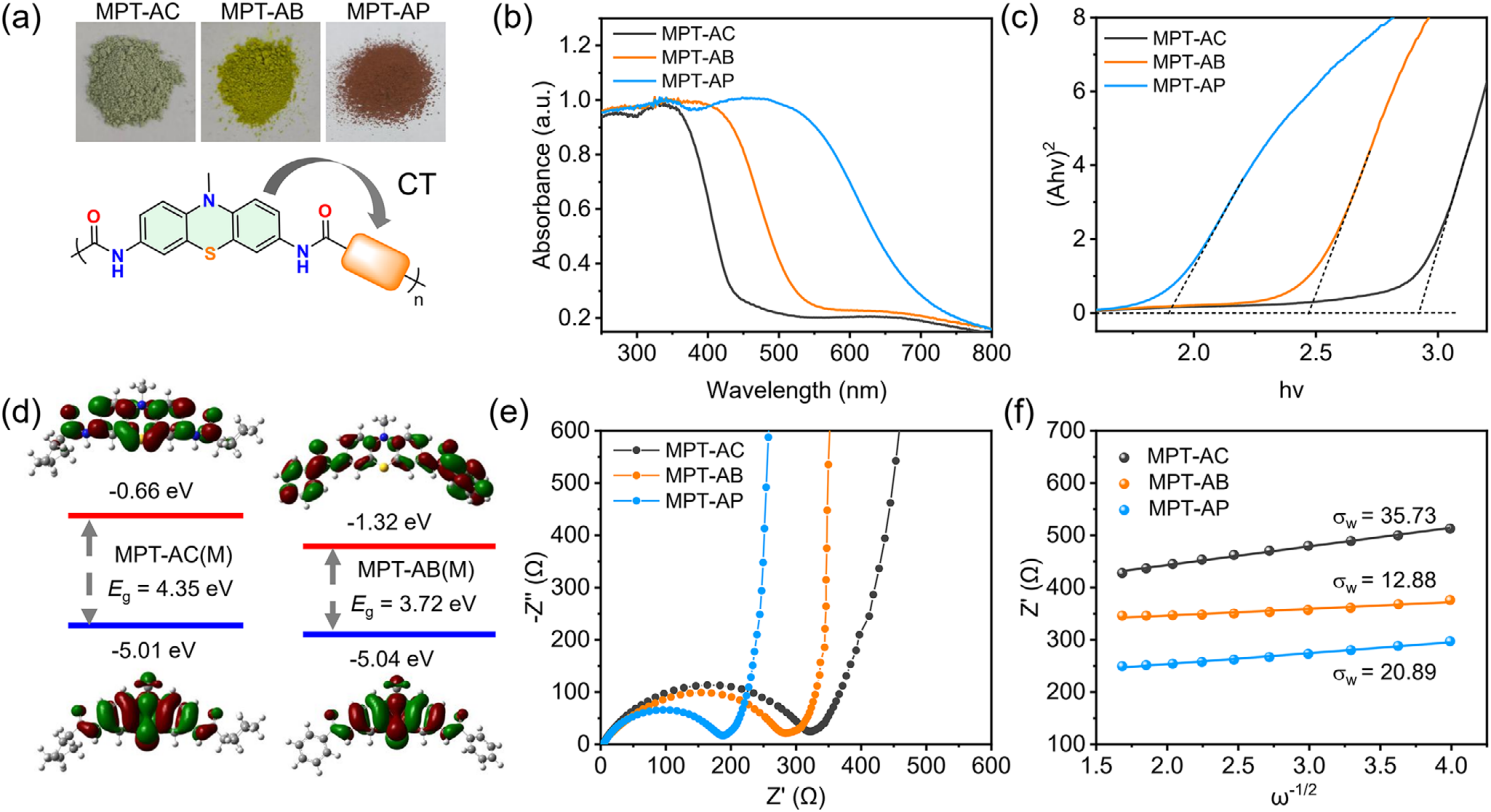
(a) Top: Digital photographs showing the colors of MPT‐AC, MPT‐AB, and MPT‐AP solids; bottom: proposed charge transfer from phenothiazine donor to linker. (b) UV–vis spectra and (c) corresponding Tauc plots of MPT‐AC, MPT‐AB, and MPT‐AP in the solid state. (d) HOMO and LUMO orbitals for MPT‐AC and MPT‐AB, as well as their calculated energies, calculated at the B3LYP/6‐31G(d, p) level. (e) Nyquist plots and (f) relationships between Z and ω^−1/2^ of MPT‐AC, MPT‐AB, and MPT‐AP electrodes.

Among the three polymers, **MPT‐AB** with the benzene linker exhibits the best battery performance in terms of cycling stability and delivered capacity, outperforming many reported phenothiazine‐based cathodes (Table S2). To elucidate the influence of the linkages on electrochemical performance, the ionic and electronic conductivities, two key factors determining battery performance, were examined. The three polymers exhibit distinct solid‐state colors (off‐white, yellow, and red; Figure 5a), highlighting the crucial role of the linkers in modulating the optical bandgaps. Figure 5b presents the solid‐state UV–vis diffuse reflectance spectra of the three polymers. A clear redshift is observed across the series, with absorption edges extending to approximately 500, 600, and 800 nm for **MPT‐AC**, **MPT‐AB**, and **MPT‐AP**, respectively. The optical bandgaps (*E*_g_) estimated from Tauc plots follow the order: **MPT‐AC** (2.92 eV) > **MPT‐AB** (2.47 eV) > **MPT‐AP** (1.89 eV), as shown in Figure 5c. The stepwise narrowing of the optical bandgap with increasing electron‐accepting strength of the linkers can be attributed to enhanced through‐space and/or through‐bond charge transfer.

The same trend in *E*_g_ is further confirmed by DFT calculations. Using one repeat unit of each polymer, denoted MPT‐AC(M), MPT‐AB(M), and MPT‐AP(M), their frontier molecular orbital diagrams and energy levels are shown in Figure 5d and Figure S11. As the electron‐withdrawing ability of the linker progressively increases, the LUMO energies are dramatically lowered, while the HOMO energies remain only slightly altered. This results in a narrowing of the *E*_g_ and facilitates charge transfer from the MPT unit to the linkers. In general, a narrower bandgap indicates more extensive electron delocalization and thus implies better electronic conductivity.

Electrochemical impedance spectroscopy (EIS) provided further insights into the interfacial charge dynamics in electrodes. The Nyquist plots reveal a semicircle at high frequencies, which is associated with the charge‐transfer resistance (*R*_CT_), denoted by the diameter of the semicircle. The *R*_CT_ varies considerably among the three materials: **MPT‐AC**: *R*_CT_ = 321 Ω; **MPT‐AB**: *R*_CT_ = 282 Ω; **MPT‐AP**: *R*_CT_ = 189 Ω (Figure 5e). A lower *R*_CT_ value suggests better electronic conductivity. Moreover, the Warburg coefficients (*σ*_w_) extracted from the low‐frequency inclined lines in Figure 5f were determined to be 35.73 for **MPT‐AC**, 12.88 for **MPT‐AB**, and 20.89 for **MPT‐AP**. A lower *σ*_w_ value indicates a higher ion diffusion coefficient (*D*). Among the three polymer electrodes, **MPT‐AB** exhibits the largest *D* value of 3.3 × 10^−11^ cm^2^ s^−1^, which is significantly higher than those of the other two (Figure S12). The best ion transport of **MPT‐AB** was also confirmed by the galvanostatic intermittent titration technique (GITT) (Figure S13). These trends indicate that increasing the electron‐withdrawing property of the linkers facilitates more efficient charge transfer within the electrode.

Collectively, linkage engineering is effective in modulating the optoelectronic properties and electron/ion transport kinetics of phenothiazine‐based polyamides, in addition to suppressing solubility. Both experimental and theoretical data demonstrate that strengthening the electron‐withdrawing nature of the linker narrows the bandgap, enhances electronic conductivity, and facilitates ionic conductivity. Notably, it may also raise the oxidation potential to a level high enough to cause decomposition, as reflected in **MPT‐AP**, which bears the strongest electron‐accepting pyrazine linker. Among the series, **MPT‐AB**, with well‐balanced optoelectronic characteristics and favorable electron/ion transport kinetics, achieves the best battery performance.

### 2.5 Structural Evolution

**FIGURE 6:**
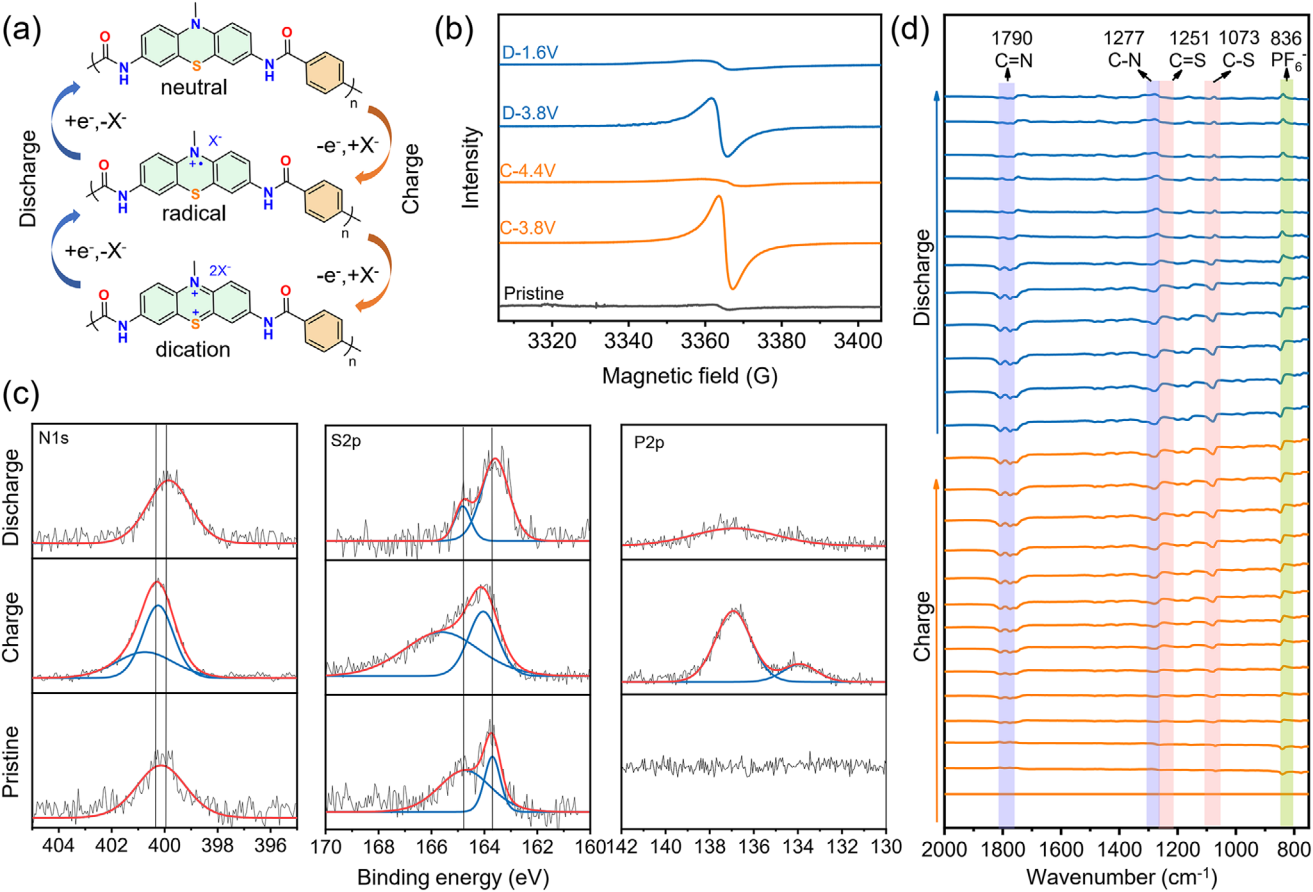
Structural evolution study of MPT‐AB. (a) Redox mechanism and structural evolution. (b) Ex situ EPR spectra recorded at different charge and discharge states. (c) Ex situ high‐resolution N 1s, S2p, and P2p XPS spectra in the pristine, fully charged, and fully discharged states. (d) In situ FT–IR spectra at different states of charge and discharge.

To gain a deeper understanding of the structural evolution of the **MPT‐AB** cathode during cycling, a series of ex situ and in situ characterization techniques were performed at various charge and discharge states. Figure 6a illustrates the proposed structural evolution of **MPT‐AB**, which involves two distinct redox events. The redox center of **MPT‐AB** reversibly cycles through three states: neutral, radical intermediate, and dicationic species, during charge and discharge. This structural evolution is coupled with the insertion and deinsertion of anions to offset the positive charge.

Given that the redox process generates radical cation intermediates, ex situ electron paramagnetic resonance (EPR) experiments were conducted, as shown in Figure 6b. As expected, the pristine **MPT‐AB** cathode exhibited no noticeable EPR signal due to its neutral state. Upon charging to 3.8 V, a strong EPR signal at 3365 G emerged, indicating partial oxidation of the phenothiazine units and the generation of radical intermediates. This signal diminished upon further charging to 4.4 V, confirming complete two‐electron oxidation. During the subsequent discharge process, the EPR signal intensity reversed its trend, demonstrating the electrochemical reversibility of the process.

Ex situ x‐ray photoelectron spectroscopy (XPS) was employed to probe the valence state evolution of **MPT‐AB** during cycling (Figure 6c). For the two core elements, nitrogen and sulfur, in the phenothiazine moiety, a shift to higher binding energies was observed in the charged electrode, reflecting a decrease in electron density due to oxidation. Specifically, the N 1s XPS spectrum of the charged **MPT‐AB** cathode exhibited a distinct C═N^+^ peak at 400.7 eV. Owing to the more diffuse nature of the sulfur nucleus and its exclusive presence in the phenothiazine ring, the S 2p XPS spectrum showed a more pronounced shift to higher binding energies, with peaks appearing at 165.64 and 164.04 eV in the charged state. These XPS spectral features were recovered during the subsequent discharge process, indicating good electrochemical stability. As for the P 2p XPS spectrum, no signal was detected for the pristine **MPT‐AB** cathode. However, upon charging to 4.4 V, two intense peaks emerged at 136.94 and 133.94 eV, revealing the insertion of PF_6_^−^ anions into the cathode during charging. These peaks significantly diminished when the electrode was discharged to 1.6 V, suggesting the release of PF_6_^−^ in the discharged state.

Additionally, in situ FT‐IR analysis provides further insight into the structure transformation and anion storage mechanism (Figure 6d). The initial state was set as the background to reveal changes during cycling. A decrease in the FT–IR signal indicates weakening, while an increase reflects the formation of new functional groups. During the charge process, a gradual increase in two new peaks at 1790 and 1251 cm^−1^ corresponding to the vibration of the C═N and C═S is observed. Meanwhile, the peak intensities of C─N and C─S bonds at 1277 and 1073 cm^−1^ gradually decrease. In addition, a gradual increase in the intensity of the peak at 836 cm^−1^ is observed, corresponding to the characteristic vibration of the PF_6_^−^ anion, indicative of anion insertion. During the subsequent discharging process, the above FT‐IR spectra change are reversed, again demonstrating the excellent electrochemical stability.

### 2.6 Full Battery

**FIGURE 7:**
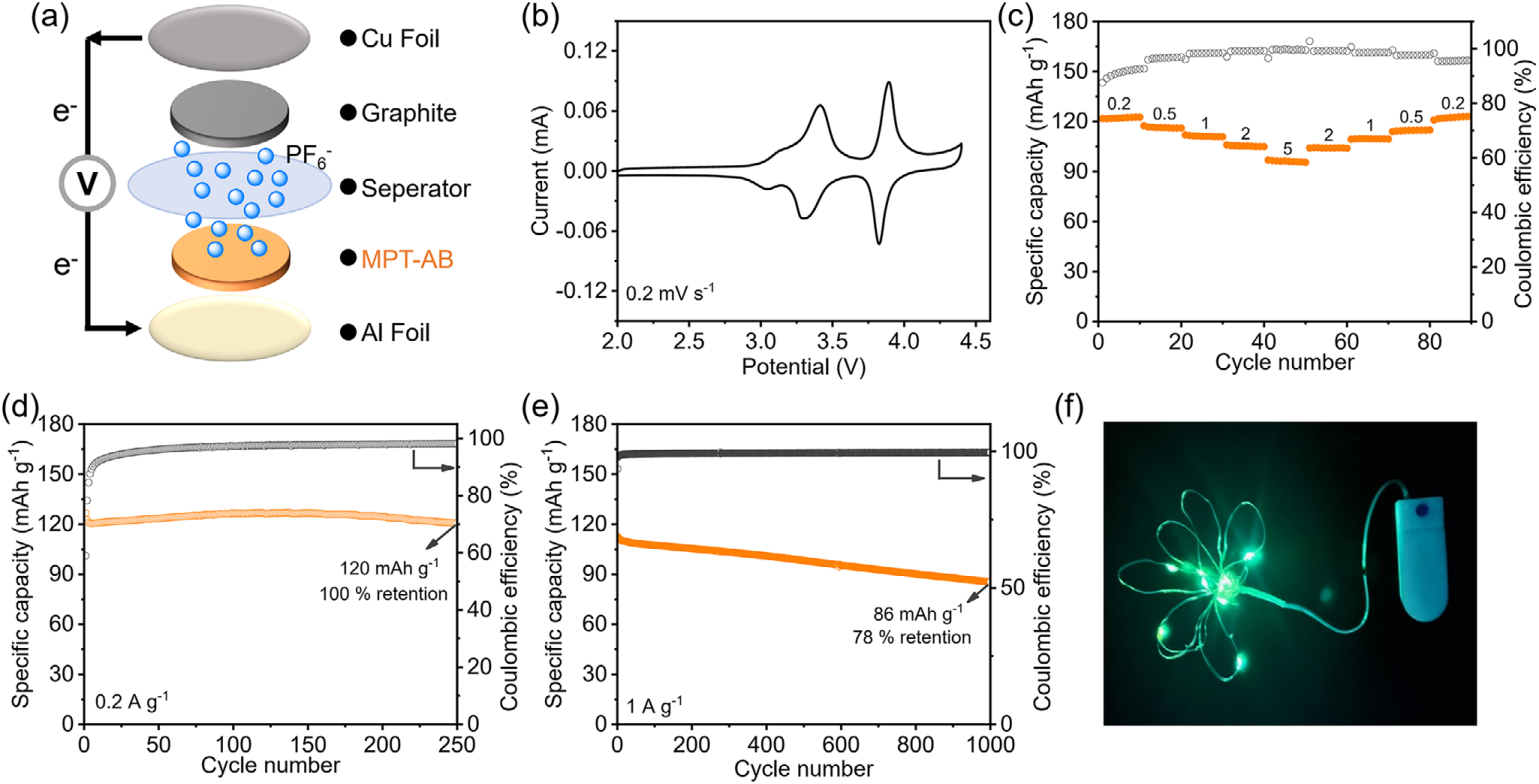
Performance of MPT‐AB//graphite full battery. (a) Schematic diagram of the as‐fabricated full battery. (b) CV curve, (c) rate performance, and (d, e) cycling performance of MPT‐AB//graphite full battery. (f) Optical image showing a full battery device powering an LED bulb.

The excellent performance of the **MPT‐AB** cathode in half‐cell configurations inspired us to assemble a full cell to further explore its practical applications. Pre‐lithiated graphite was selected as the anode. Figure 7a presents a schematic diagram of the **MPT‐AB**//graphite full cell. Similar to its half cell, the full cell exhibited two redox pairs in its CV curves (Figure 7b) and two corresponding plateaus in the charge–discharge curves (Figure S14). However, the output voltages were slightly lower by ca. 0.1 V, which is attributed to the higher redox potential of graphite compared to that of Li metal. The full cell delivered a specific capacity of 123 mAh g^−1^ at 0.2 A g^−1^ and achieved a rate capability of 97 mAh g^−1^ at 5.0 A g^−1^ (Figure 7c). When the current density was restored to 0.2 A g^−1^, the specific capacity was fully recovered, demonstrating excellent reversibility. Impressively, the **MPT‐AB**//graphite full cell exhibited outstanding cycling stability, delivering a capacity of 120 mAh g^−1^ with 100% capacity retention after 250 cycles at 0.2 A g^−1^, and a capacity of 86 mAh g^−1^ with 78% capacity retention after 1000 cycles at 1 A g^−1^ (Figure 7d,e). Additionally, the full cell successfully lights up a loop of LED bulbs (Figure 7f), highlighting its potential for practical applications.

## 3 Conclusions

In summary, three phenothiazine‐based polyamides featuring electronically distinct linkages have been designed and utilized as a platform to investigate the impact of linkage engineering on their electrochemical properties and electron/ion transport kinetics. Enhancing the electron‐withdrawing character of the linkers presents a double‐edged sword: while it improves charge transfer, narrows the bandgap, and promotes both electronic and ionic conduction, it can also elevate the oxidation potential to a critical level, thereby limiting the accessibility of the full reversible redox process. Among the series, **MPT‐AB,** incorporating a moderately electron‐withdrawing benzene linker, achieves an optimal balance, enabling a reversible two‐step redox process along with faster ion and electron transport, which collectively contribute to superior LOB performance. Specifically, the **MPT‐AB** cathode delivers a high reversible capacity of 150 mAh g^−1^ at 0.2 A g^−1^, a satisfactory rate capability (103 mAh g^−1^ at 5 A g^−1^), and long‐term cycling stability with 90 % and 72% capacity retention after 2000 and 5000 cycles at 5 A g^−1^. These findings underscore the effectiveness of strategic linkage engineering in phenothiazine‐based polyamides for enhancing battery performance, thereby facilitating their practical application in lithium‐ion batteries.

## Author Contributions

**Shujuan Cao**: methodology, formal analysis, investigation. **Chunping Ren**: validation, data curation. **Jing Qin**: validation, data curation. **Xiujuan Wang**: supervision, data curation. **Xiaoming He**: conceptualization, methodology, investigation, funding acquisition, supervision, writing – review and editing, project administration.

## Funding

This work was supported by the Fundamental Research Funds for the Central Universities National (GK202201006), the Innovation Capability Support Program of Shaanxi (No. 2020TD024), and the Shaanxi Provincial Key Research and Development Program Project (No. 2026CY‐YBXM‐576).

## Conflicts of Interest

The authors declare no conflicts of interest.

## Supporting information




**Supporting File**: advs77960‐sup‐0001‐SuppMat.pdf.

## Data Availability

The data that support the findings of this study are available from the corresponding author upon reasonable request.
