# Supplementary material for: Linkage Engineering Boosts Battery Performance of Two‐Electron Phenothiazine‐Based Polyamide Cathodes
Source: Adv Sci (Weinh). 2026 Sep 27:e77960. Online ahead of print. doi: 10.1002/advs.77960 (PMC13616290; doi:10.1002/advs.77960)
Supplement: Supplementary file 1 [file ADVS-9999-e77960-s001.pdf]

Supporting Information

Linkage Engineering Boost Battery Performance of Two-Electron Phenothiazine Based Polyamide Cathodes

*Shujuan Cao, Chunping Ren, Jing Qin, Xiujuan Wang, Xiaoming He**

*To whom correspondence should be addressed

Key Laboratory of Applied Surface and Colloid Chemistry (Ministry of Education), School of Chemistry and Chemical Engineering, Shaanxi Normal University, Xi'an 710119, P.R. China

*Corresponding Author Email: xmhe@snnu.edu.cn

Experimental Section

1. Materials and Instruments

N-methyl phenothiazine, 1,4-cyclohexanedicarboxybic acid, 1,4-benzenedicarboxylic acid and 2,5-pyrazinedicarboxylic acid were purchased from Adamas. Polytetrafluoroethylene (PTFE), Ketjen Black (ECP-600J) were obtained from Shenzhen Kejing Zhida Technology Co., Ltd. The lithium chip, CR2032 battery case, 1 M $LiPF_6$ in EC:DMC:EMC (1:1:1, v/v), aluminum foil and separator (Celgard2400) were purchased from DoDoChem. Ketjen Black (ECP-600J) were dried at 80 ^{o}C for 12 h under vacuum oven before use. All other reagents or solvents were used without further purification, unless indicated otherwise.

^{1}H NMR spectra were obtained on 600 MHz BRUKER spectrometer. Mass spectrometry data were collected on a Bruker maxis UHR-TOF mass spectrometer. IR spectra were obtained on a PE-Frontier instrument. Scanning electron microscope (SEM) images were recorded using a Hitachi SU8220 system. Powder X-ray diffraction (PXRD) patterns were recorded on a Bruker D8 Advance instrument with Cu-Kα radiation. Thermogravimetric analysis (TGA) was performed under nitrogen atmosphere from 25 to 800 °C with a heating rate of 10 °C min^{-1}. The X-ray photoelectron spectra (XPS) experiments were carried out on a PHI-5400 electron spectrometer. Diffuse reflectance spectra were collected on a UV–vis spectrophotometer with integrating sphere (SHIMADZU UV-2600). Theoretical calculations were carried out at the B3LYP/6-31G (d, p) level by using the GAUSSIAN 09 suite of program. [S1]

2. Synthesis

2.1 Synthesis of MPT-A.

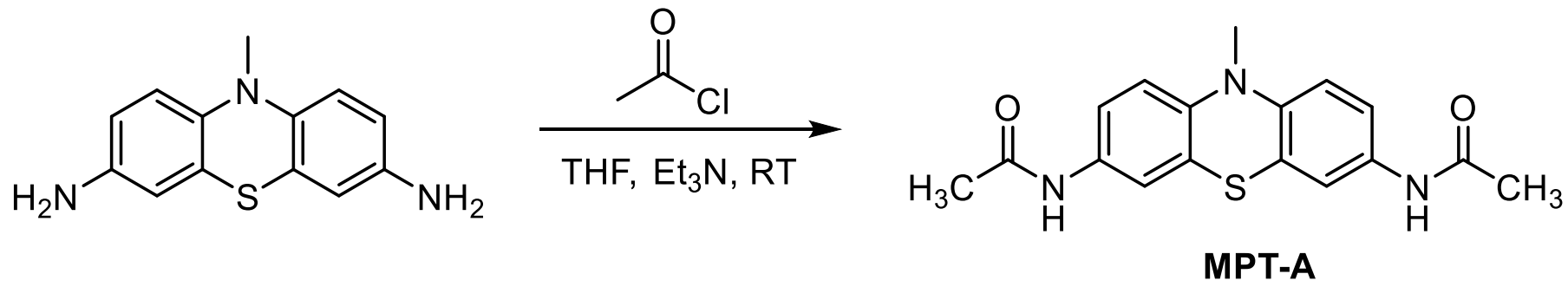

3,7-diamino-N-methyl phenothiazine ($MPT-NH_2$) was prepared according to our previous work (*Adv. Energy Mater.* 2025, 15, 2403029).

To a solution of MPT-NH_2 (0.5 g, 2.06 mmol) and Et_3N (1.2 mL, 0.84 g, 8.3 mmol) in THF (25 mL) was added acetyl chloride (0.65 g, 8.3 mmol) at 0 ºC. The reaction was stirred overnight at room temperature, and then poured into water (100 mL). The product was collected by filtration and washed with water and methanol. Yield: 0.62 g, 92 %. ^{1}H NMR (600 MHz, DMSO-d_6): δ/ppm = 9.93 (s, 2H; NH), 7.54 (s, 2H; ArH), 7.39 (d, J = 9.0 Hz, 2H; ArH), 6.92 (d, J = 8.4 Hz, 4H; ArH), 3.30 (s, 3H; NCH_3), 2.06 (s, 6H; CH_3). ^{13}C NMR (150 MHz, DMSO-d_6): δ/ppm = 168.38, 141.24, 134.72, 122.16, 118.80, 117.90, 114.71, 35.41, 24.33. HR-MS: m/z calculated for $C_{17}H_{18}N_3O_2S$ $[M+H]^+$ 328.1114, found 328.1116.

2.2 General synthesis of MPT-AC, MPT-AB and MPT-AP

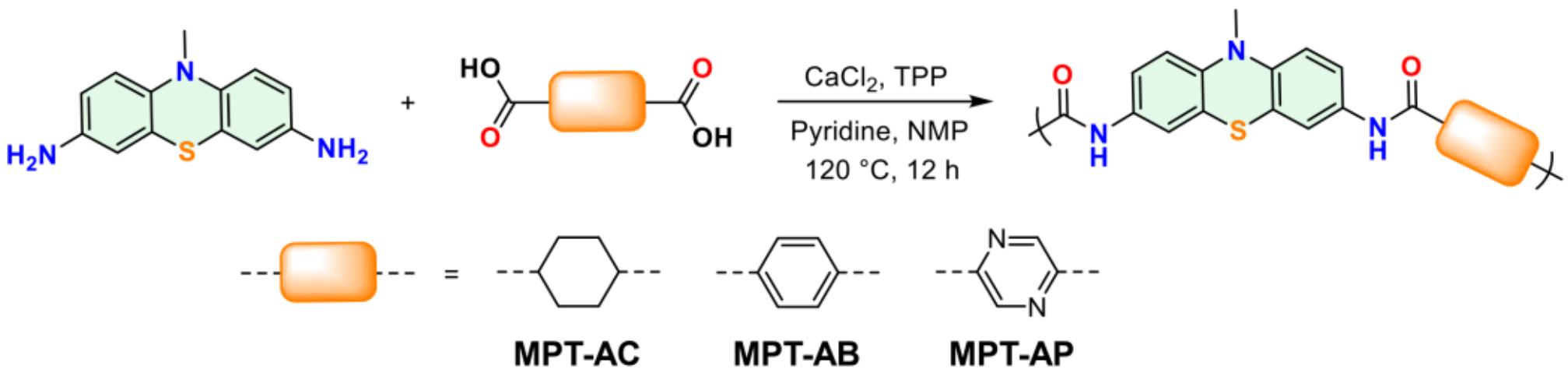

A mixture of MPT-NH_2 (145.8 mg, 0.6 mmol), diacid monomer (0.6 mmol), anhydrous calcium chloride (100 mg), triphenyl phosphite (0.6 mL), pyridine (0.3 mL), and NMP (1 mL) was heated with stirring at 120 °C for 12 h. After cooling down to room temperature, the resulting polymer solution was poured slowly into methanol (ca. 30 mL) giving rise to a precipitate. The precipitated product was collected by filtration, washed thoroughly with DMF, hot water and methanol, followed by dry under vacuum. The diacid monomers used for preparing MPT-AC, MPT-AB and MPT-AP are 1,4-cyclohexanedicarboxybic acid, 1,4-benzenedicarboxylic acid, 2,5-pyrazinedicarboxylic acid, respectively. The yields for MPT-AC, MPT-AB and MPT-AP are close to 100 %.

3. Solubility Test of MPT and MPT-A

To 50 mg MPT or 1 mg MPT-A, EC/DMC/EMC (1:1:1, v/v/v) electrolyte was gradually added in 20-100 µL portions at 5-minute intervals until the materials were fully dissolved.

4. Battery Fabrication and Testing

The working electrodes were prepared by mixing the active polymer (MPT-AC, MPT-AB or MPT-AP), Ketjen Black (ECP-600J) and PTFE with a mass ratio of 6/3/1 (unless other noted) using *i*PrOH

as dispersion solvent. Then the slurry was coated on the aluminum foil with a diameter of 9 mm and the mass loading of the active materials was *ca.* 0.6-0.8 mg cm^{-2}. The fully prepared electrodes were then dried at 80 °C overnight in a vacuum oven in order to remove any residual solvent. Assembly of the CR2032-type coin-type half cells were carried out in argon-filled glove box (<0.01 ppm of oxygen and water), with lithium metal as the anode, polypropylene separator film (Celgard2400) as the separator, and 1 M $LiPF_6$ in EC/DMC/EMC (1:1:1, v/v/v) as the electrolyte. The CV and EIS tests were performed an CHI760E electrochemical working station, and the charge-discharge curves were conducted on LAND CT2001A at 30 °C, unless otherwise noted. Galvanostatic charge and discharge (GCD) and galvanostatic intermittent titration technique (GITT) tests were carried out using LANHE-CT2001A system (Wuhan, China) in the potential ranging from 1.6 to 4.4 V (vs. Li/Li^+) at different current densities. GITT was conducted at 0.05 A g^{-1} in the potential range of 1.6-4.4 V (vs. Li/Li^+) with a constant current pulse of 15 min and a relax of 30 min recorded on LANHE CT3001A at 30 °C.

Graphite||Li half battery: Graphite, conductive carbon black (ECP-600JD) and PTFE were mixed uniformly with a weight ratio of 6:3:1 and coated on the surface of Cu foil to prepare graphite half cells. The counter electrode, separator, and electrolyte were the same as MPT-AB half-cells. The typical average areal mass loading of graphite was about 1.4 mg cm^{-2}.

MPT-AB||graphite full battery: The prepared MPT-AB electrodes were cycled 5 times and then charged to 4.4 V to obtain the pre-charged MPT-AB electrodes while the prepared graphite electrodes were discharged to 0.005 V to obtain the pre-lithiated graphite electrodes. MPT-AB||graphite full battery was assembled using a pre-lithiated graphite electrode (discharged to 0.01 V vs. Li^+/Li) as the anode and a pre-charged MPT-AB electrode (charged to 4.4 V vs. Li^+/Li) as the cathode. The MPT-AB||graphite full cell was assembled in this work with a N/P (negative/ positive electrode) mass ratio of around 3.5, for the purpose of cycling and capacity. About 120 μL of 1 M $LiPF_6$ in EC/DMC/EMC (1:1:1, v/v/v) was used as the electrolyte per full-cell.

5. Calculations of the Electrochemical Metrics

Theoretical capacity (C_{theor}, mAh g^{-1}) was calculated according to the equation (1):

$$C_{theor} = \frac{nF}{3.6 \times M} \qquad (1)$$

where n is the number of electrons transferred per molecules, F is the Faraday's constant (96484 C mol^{-1}), M is molecular weight of the molecules.

The b-value and capacitive contribution at a particular potential were determined as follows:

The relationship between scan rate (υ, mV s^{-1}) in a CV and the corresponding cathodic or anodic peak current (i_p, A g^{-1}) is shown in equation (2).[S2] The b-value was the slope of the log(v)-log(i_p) plots according to equation (3).

$$i_p = av^b \tag{2}$$

$$\log(i_p) = \log(a) + b\log(v) \tag{3}$$

where a and b are adjustable parameters.

Moreover, the relationship between the current at a particular potential (i(V), A g^{-1}) and the scan rate (υ, mV s^{-1}) is shown in equation (4).[S3] Solving for the values of k_1 and k_2 at each potential, we can obtain the percentage of capacitive contribution the total current (k_2v/i(V)).

$$i(V) = k_1 v^{1/2} + k_2 v \tag{4}$$

6. Calculation of apparent ion diffusion coefficients (D_{app}, cm^2 s^{-1}) from GITT

The apparent diffusion coefficients (D_{app}) are calculated by the following equation: [S4]

$$D_{app} = \frac{4}{\pi\tau}\left(\frac{V_m m_B}{SM_m}\right)^2\left(\frac{\Delta E_s}{\Delta E_t}\right)^2 \tag{5}$$

where D_{app} is the apparent ion diffusion coefficient (cm^2 s^{-1}), τ is the the duration of the current pulse (s), V_m is the molar volume of the active material (cm^3 mol^{-1}), m_B is the mass of the active material in the electrolyte (g), M_m is the molar mass of active material (g mol^{-1}), S is the contact surface area (cm^2) between electrode and electrolyte, ΔE_s is the equilibrium potential change induced by current pulse, ΔE_t is the potential variation during the constant current pulse.

7. Calculation of ion diffusion coefficients (D, cm^2 s^{-1}) from EIS

According to equation 6, the linear relationship between the real part of impedance (Z') and the reciprocal of the square root of angular frequency ($\omega^{-1/2}$) is quantitatively analyzed to obtain the ion diffusion resistance σ_W, also known as Warburg coefficient.

$$Z' = R_s + R_{ct} + \sigma_w \omega^{-1/2} \qquad (6)$$

where Z' is the real impedance in EIS data, R_S is the resistance of the electrolyte, R_{ct} is the charge transfer resistance, ω is the angular frequency in the low frequency region and σ_W is the slope for the plot of Z' vs. $\omega^{-1/2}$.

Accordingly, the ion diffusion coefficient (D) of the electrode is calculated according to the equation 7:

$$D = \frac{R^2 T^2}{2A^2 n^4 F^4 C^2 {\sigma_w}^2} \qquad (7)$$

Where R is gas constant, T is absolute temperature of the test environment, A is the area of the electrodes, n is the number of electrons transferred, F is the Faraday constant, C is concentration of ion.

8. Supplementary Figures and Tables

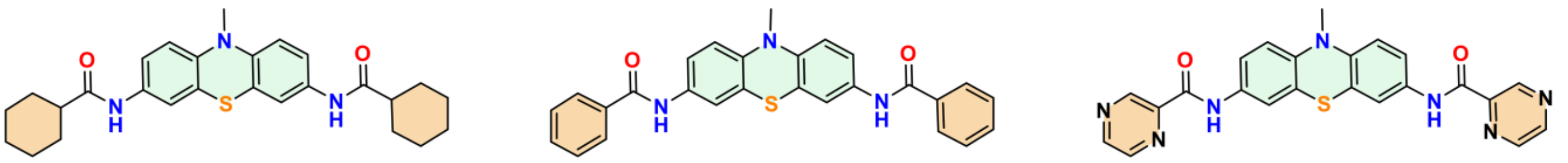

Scheme S1. Molecular structures of three polymer models, MPT-AC(M), MPT-AB(M) and MPT-AP(M) for calculation.

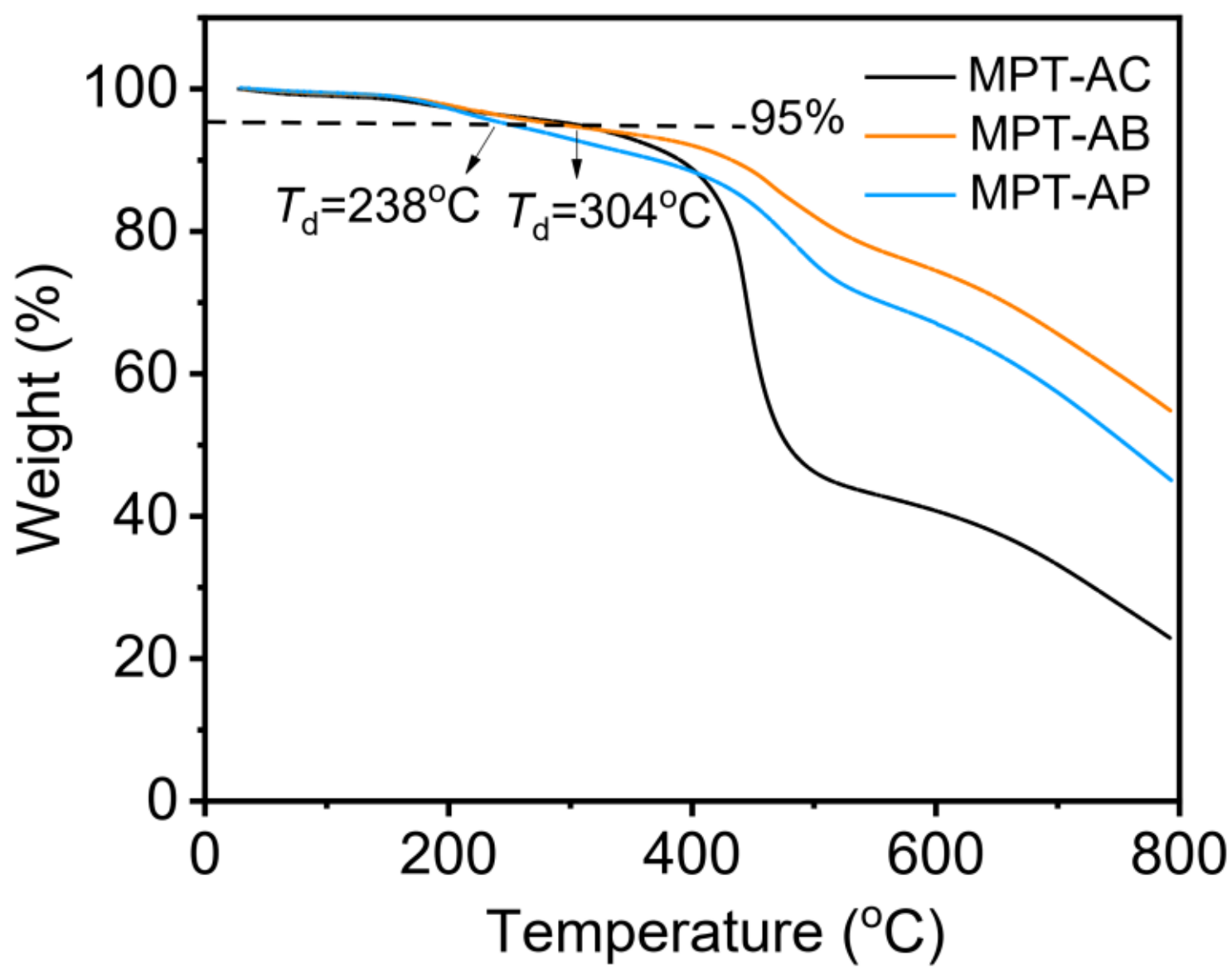

Figure S1. TGA curves of MPT-AC, MPT-AB and MPT-AP.

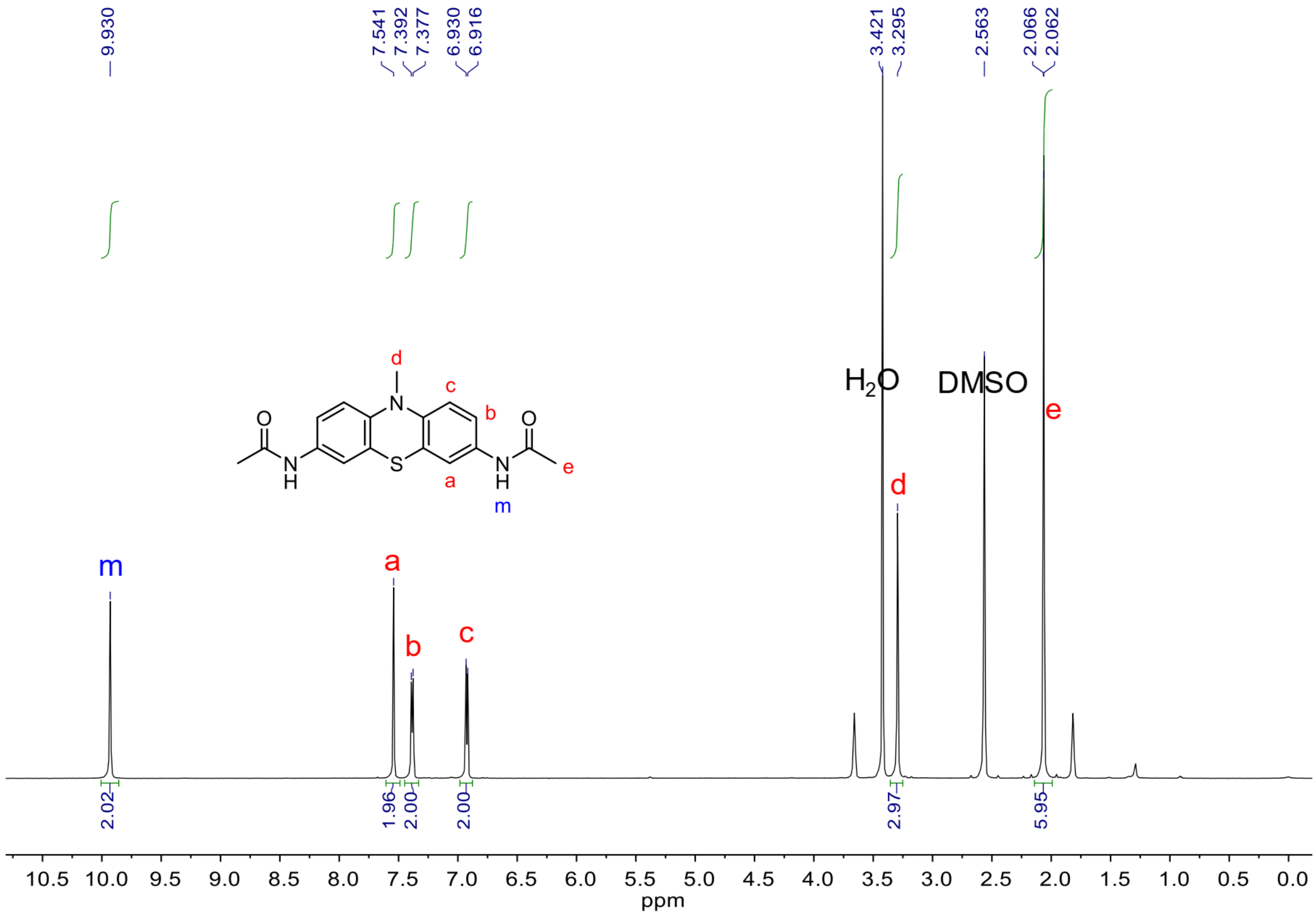

Figure S2. ^{1}H NMR spectrum of MPT-A in DMSO-d_6.

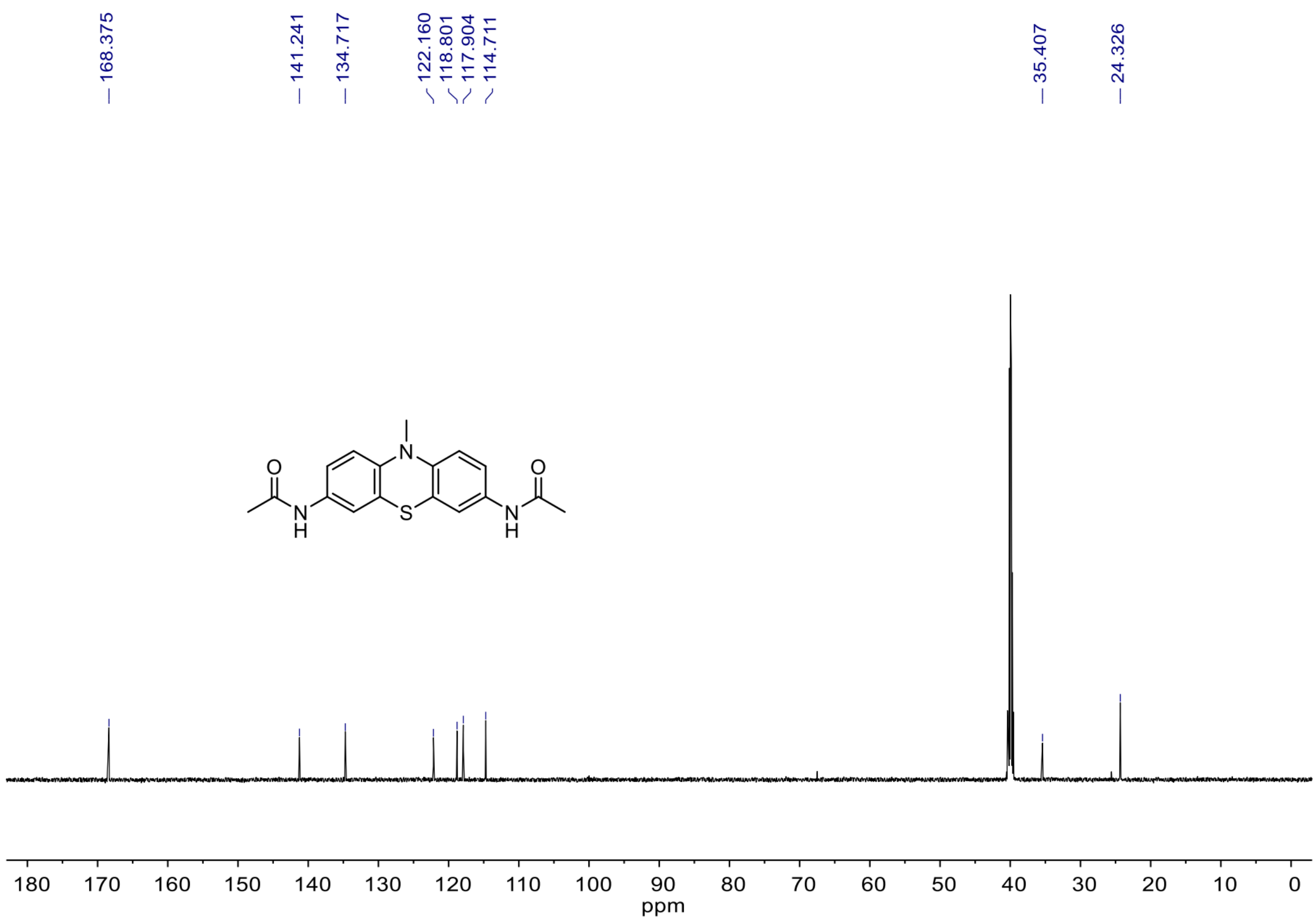

Figure S3. ^{13}C NMR spectrum of MPT-A in DMSO-d_6.

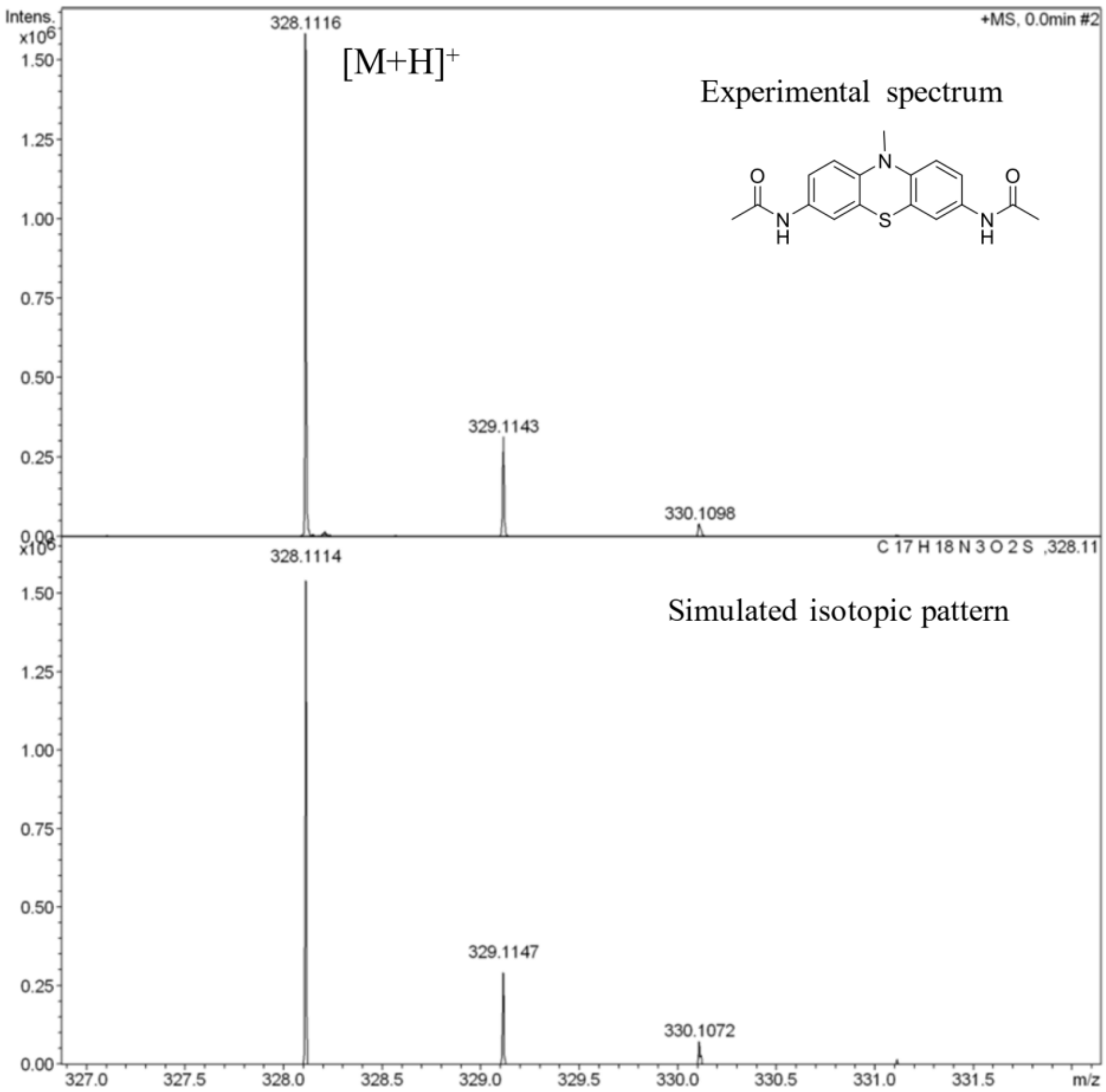

Figure S4. Mass spectrum of MPT-A.

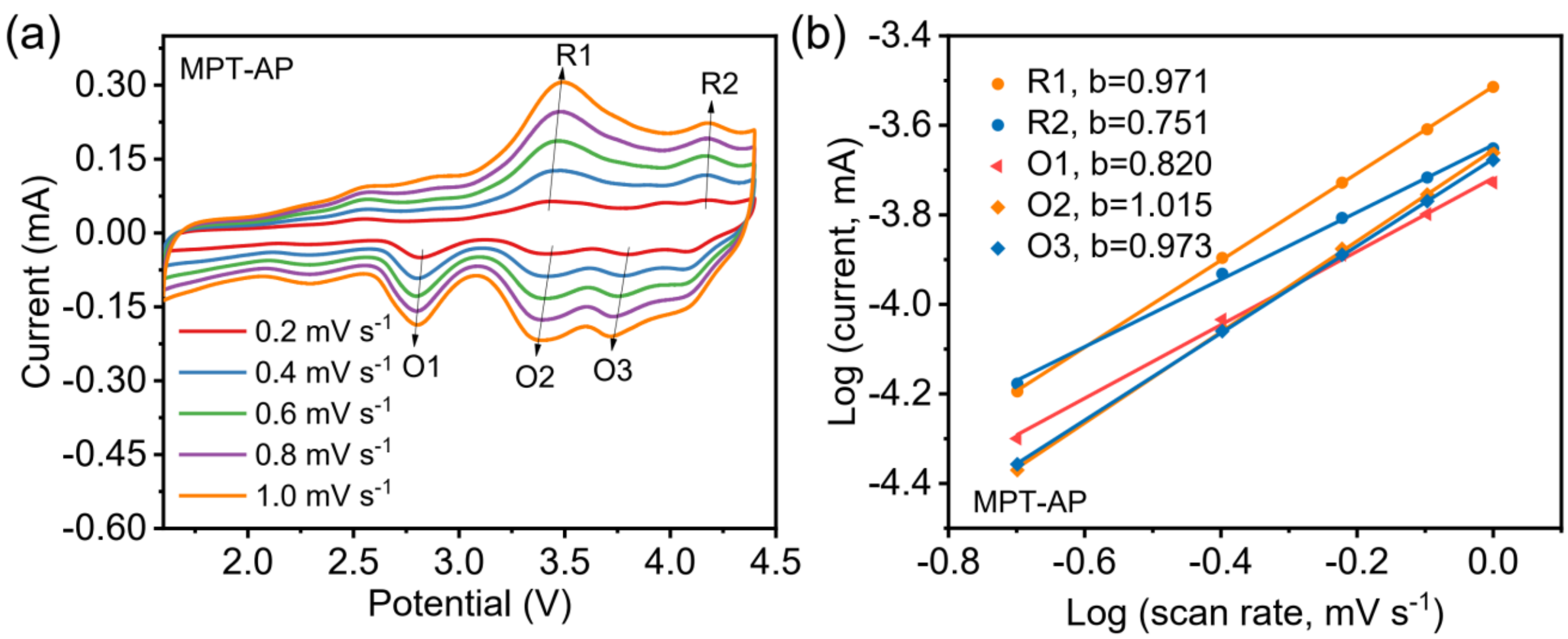

Figure S5. (a) CV curves MPT-AP electrode at different scan rates of 0.2–1.0 mV s^{-1}. (b) Plot of log(i) versus log(v) of multiple redox peaks in the CV profile of MPT-AP electrode.

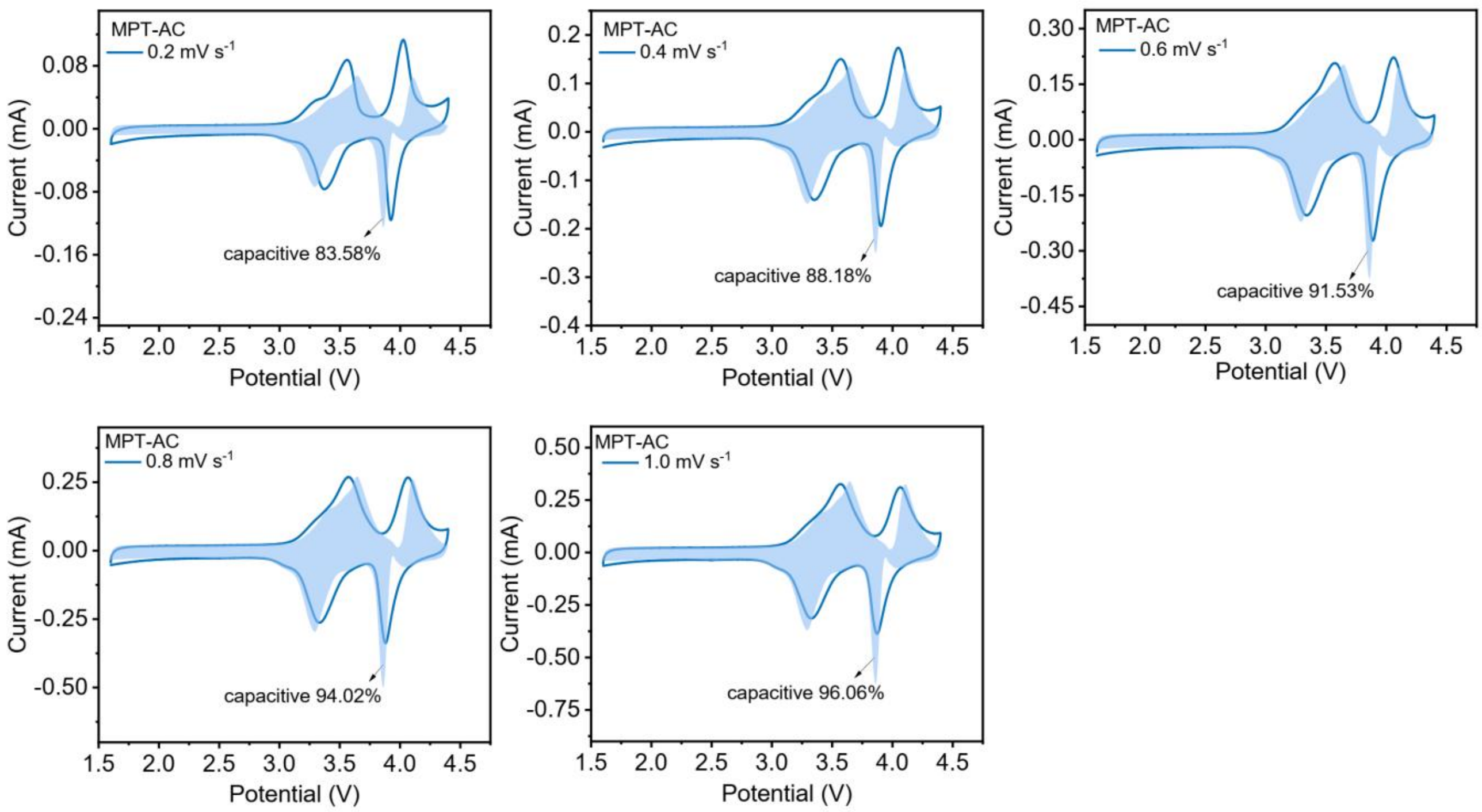

Figure S6. Capacitive contribution of MPT-AC in CV curve at different scan rates.

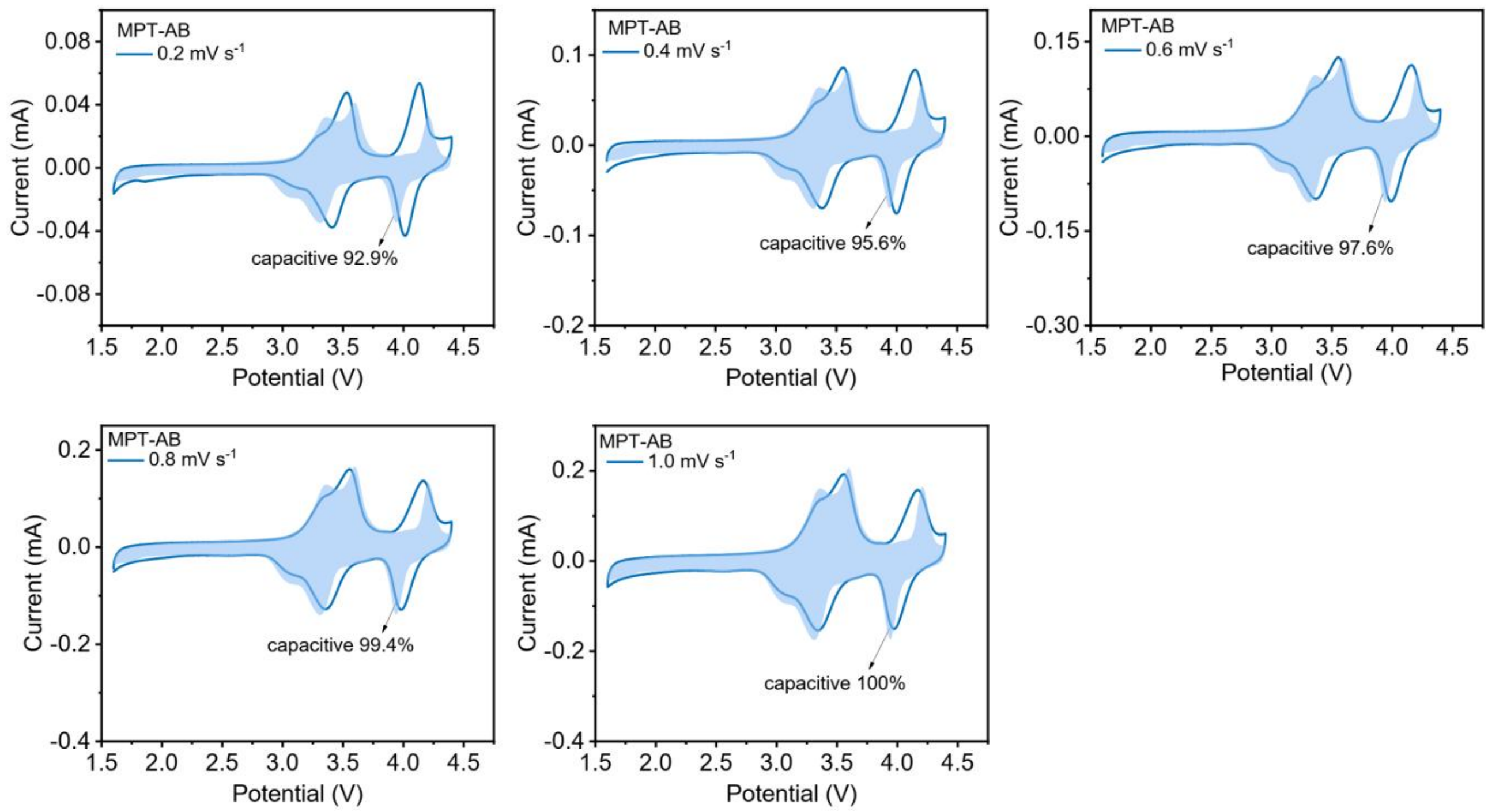

Figure S7. Capacitive contribution of MPT-AB in CV curve at different scan rates.

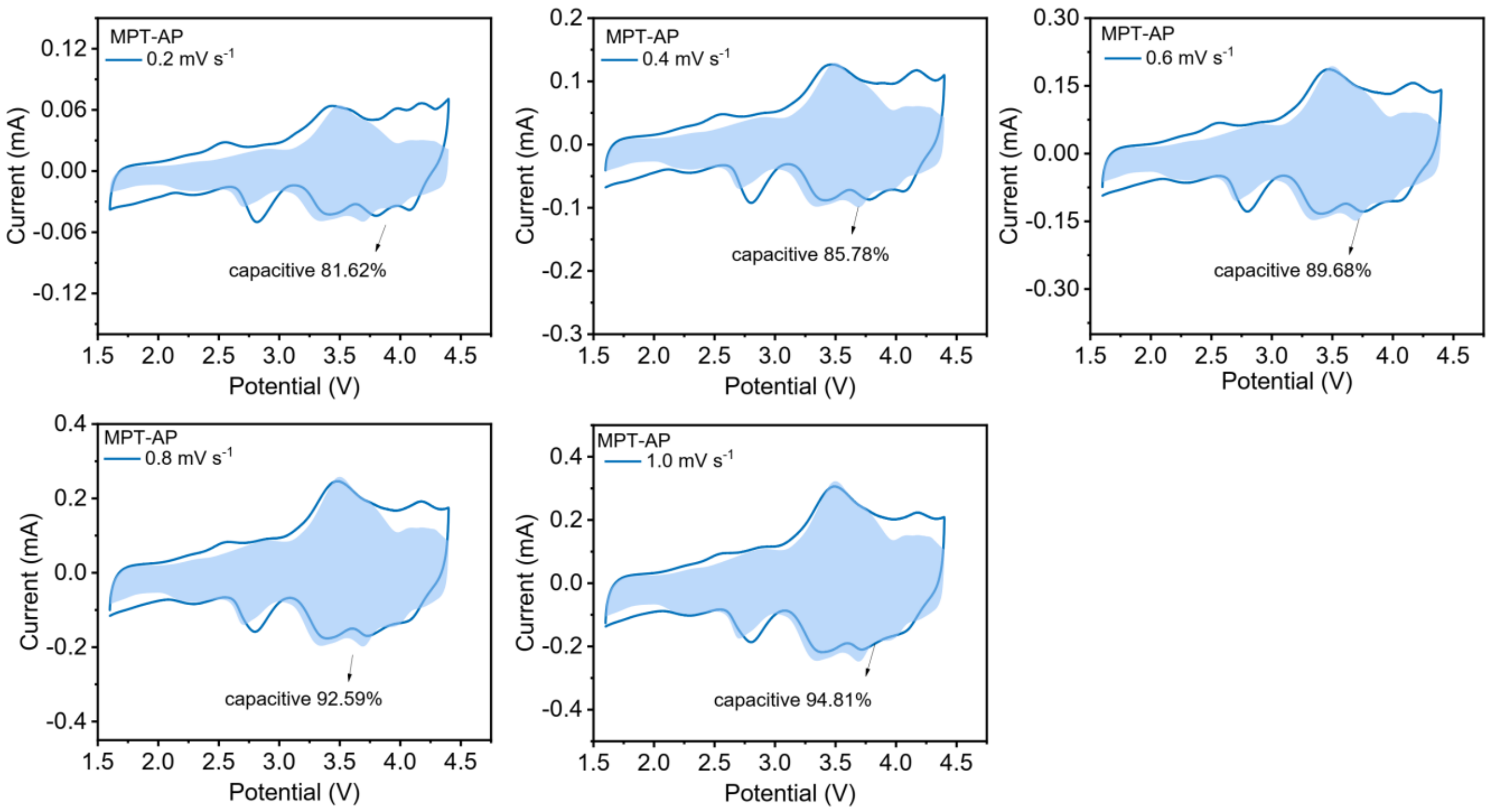

Figure S8. Capacitive contribution of MPT-AP in CV curve at different scan rates.

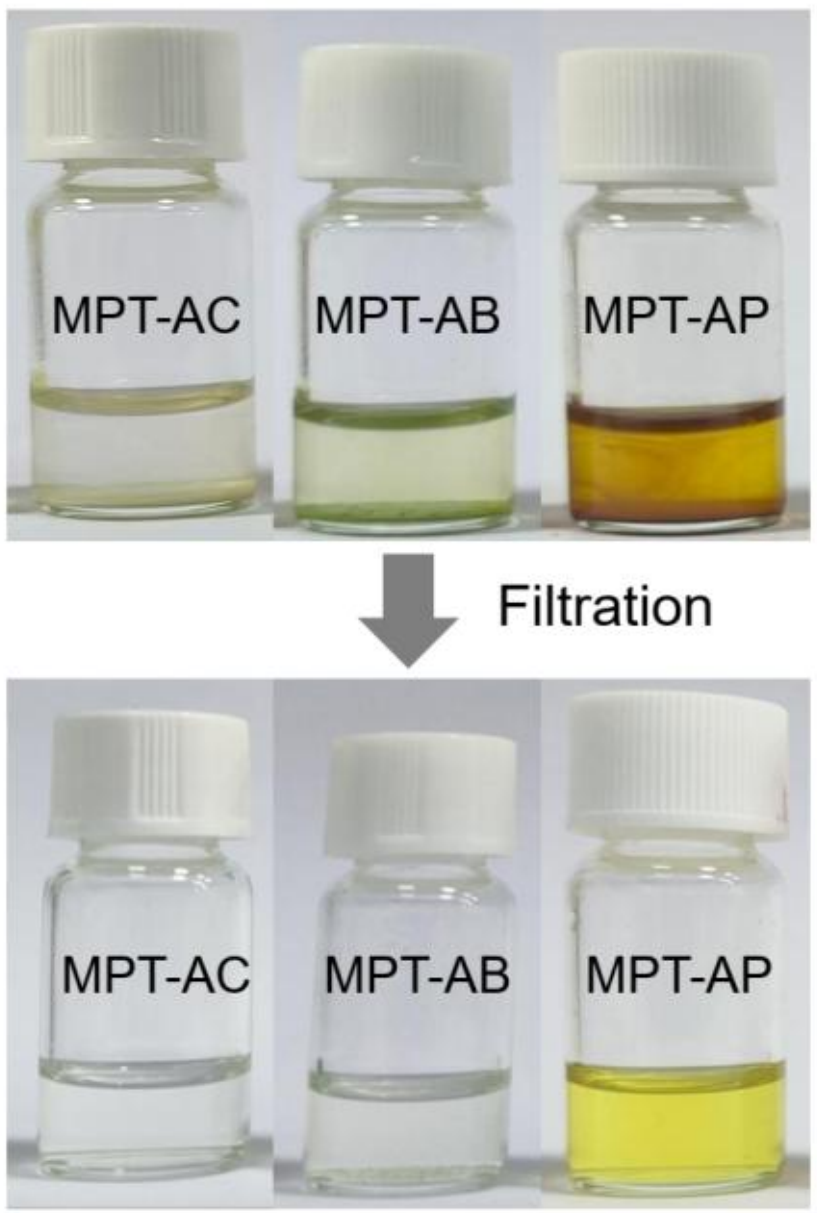

Figure S9. The photographs of MPT-AC, MPT-AB and MPT-AP in electrolyte and the corresponding filtrates.

Additional Discussion: The higher solubility of MPT-AP in the electrolyte can be tentatively ascribed to the presence of polar pyrazine functional groups, which facilitate stronger, more favorable interactions with the polar electrolyte solvent and/or promote chelation with metal ions.

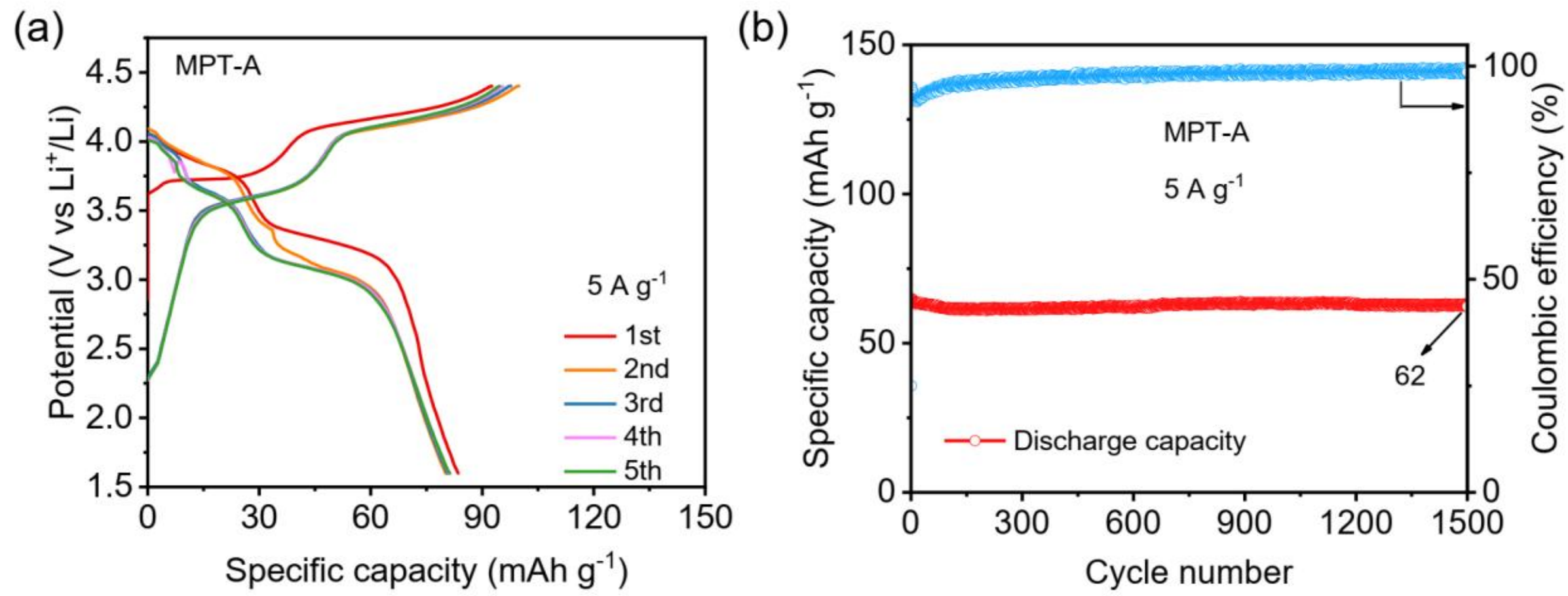

Figure S10. (a) GCD curves and (b) cycling performance of MPT-A at 5 A g^{-1}.

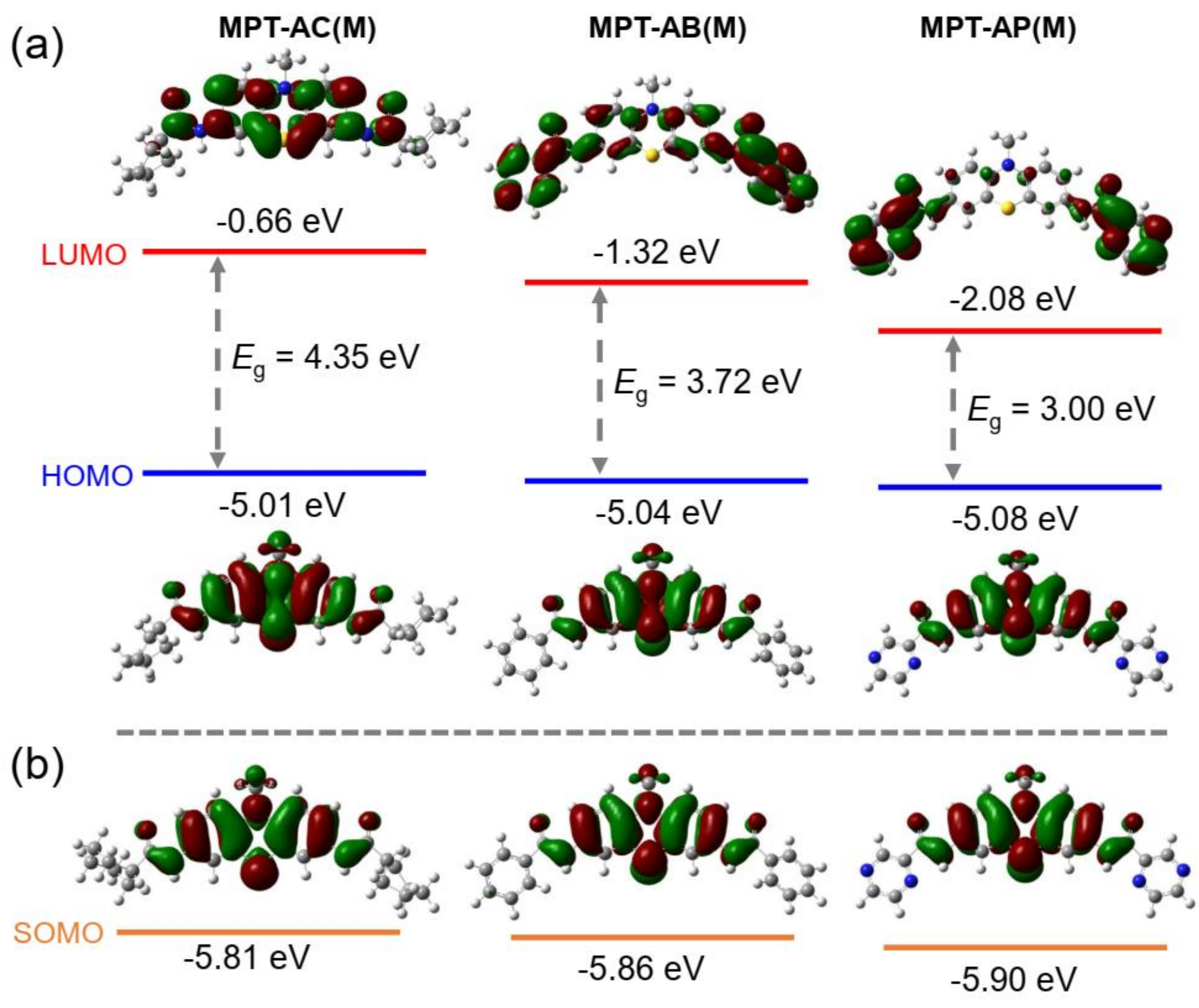

Figure S11. Frontier molecular orbital diagrams and the electronic transitions of (a) HOMO and LUMO, (b) SOMO for MPT-AC(M), MPT-AB(M) and MPT-AP(M).

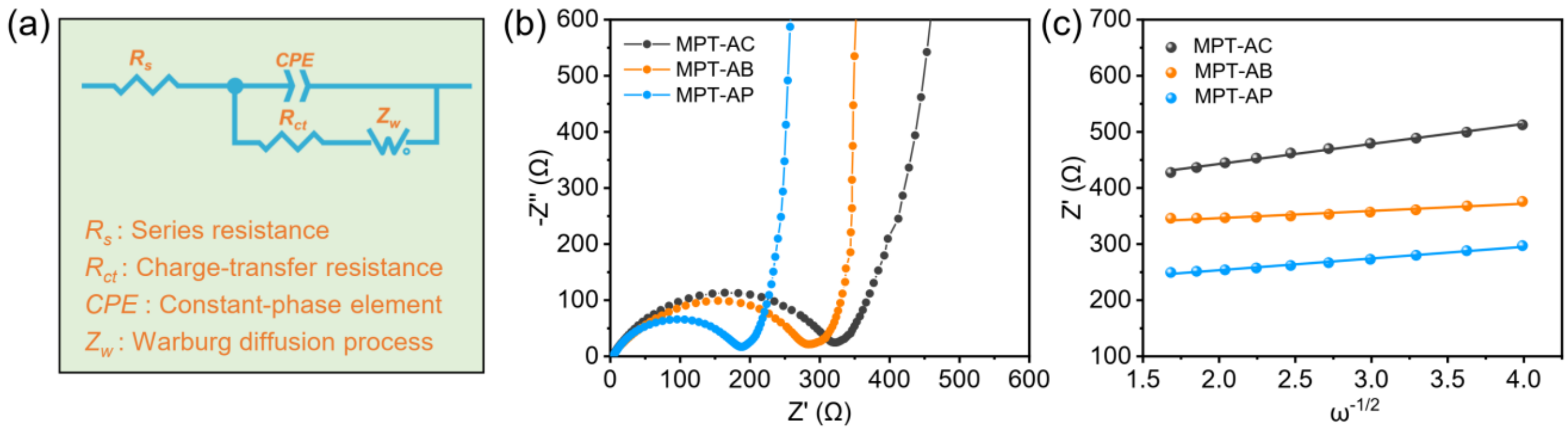

(d)

	MPT-AC	MPT-AB	MPT-AP
R_s (Ω)	1.965	18.95	1.349
R_{ct} (Ω)	321	282	189
σ_w (Ω $S^{-1/2}$)	35.73	12.88	20.89
D (cm^2 s^{-1})	4.288 × 10^{-12}	3.3 × 10^{-11}	1.25 × 10^{-11}

Figure S12. Electrochemical kinetics study. (a) Equivalent circuit used for fitting the experimental data. (b) Nyquist plots of MPT-AC, MPT-AB and MPT-AP electrode before cycling. (c) The relationship between the real part of impedance (Z') and the reciprocal of the square root of angular frequency ($\omega^{-1/2}$). (d) Summary of simulation parameters from the EIS measurements.

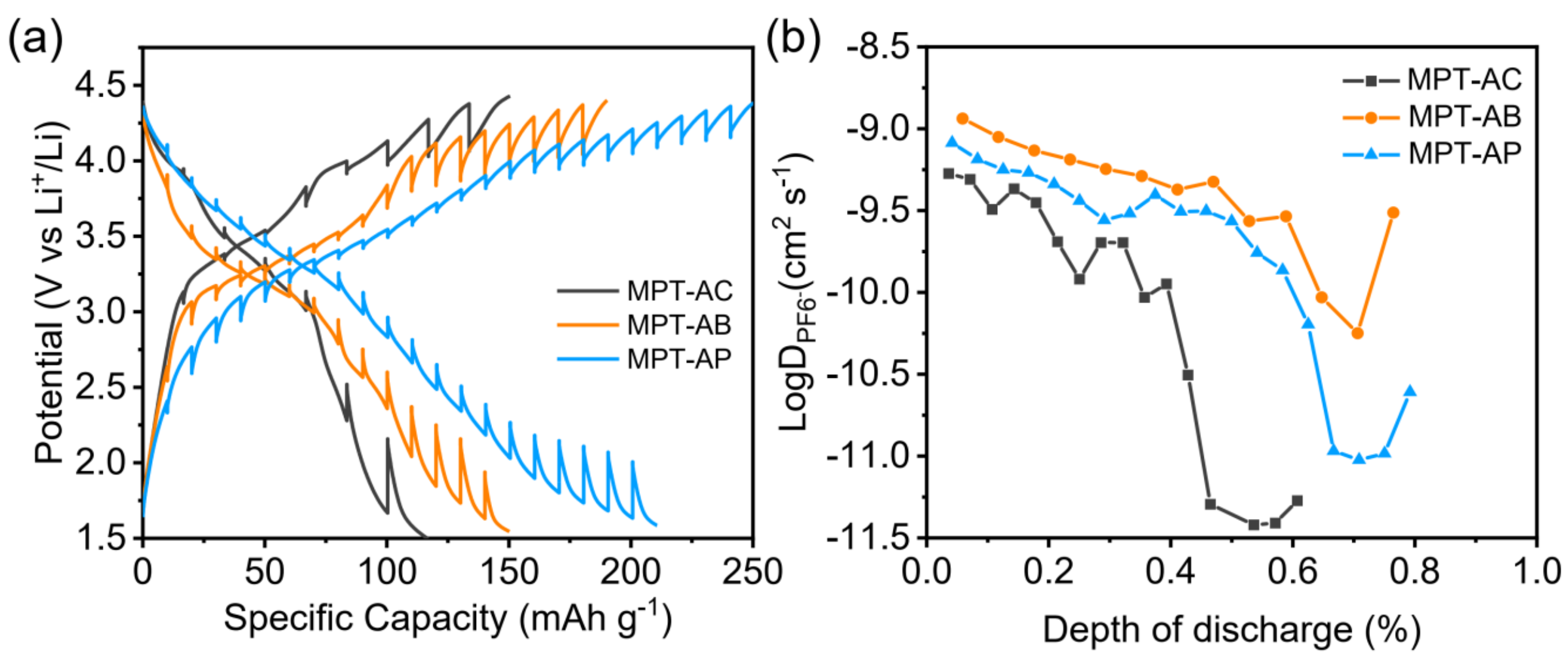

Figure S13. (a) GITT curves for MPT-AC, MPT-AB and MPT-AP electrode. (b) The PF_6^- diffusive coefficients calculated from the GITT profiles.

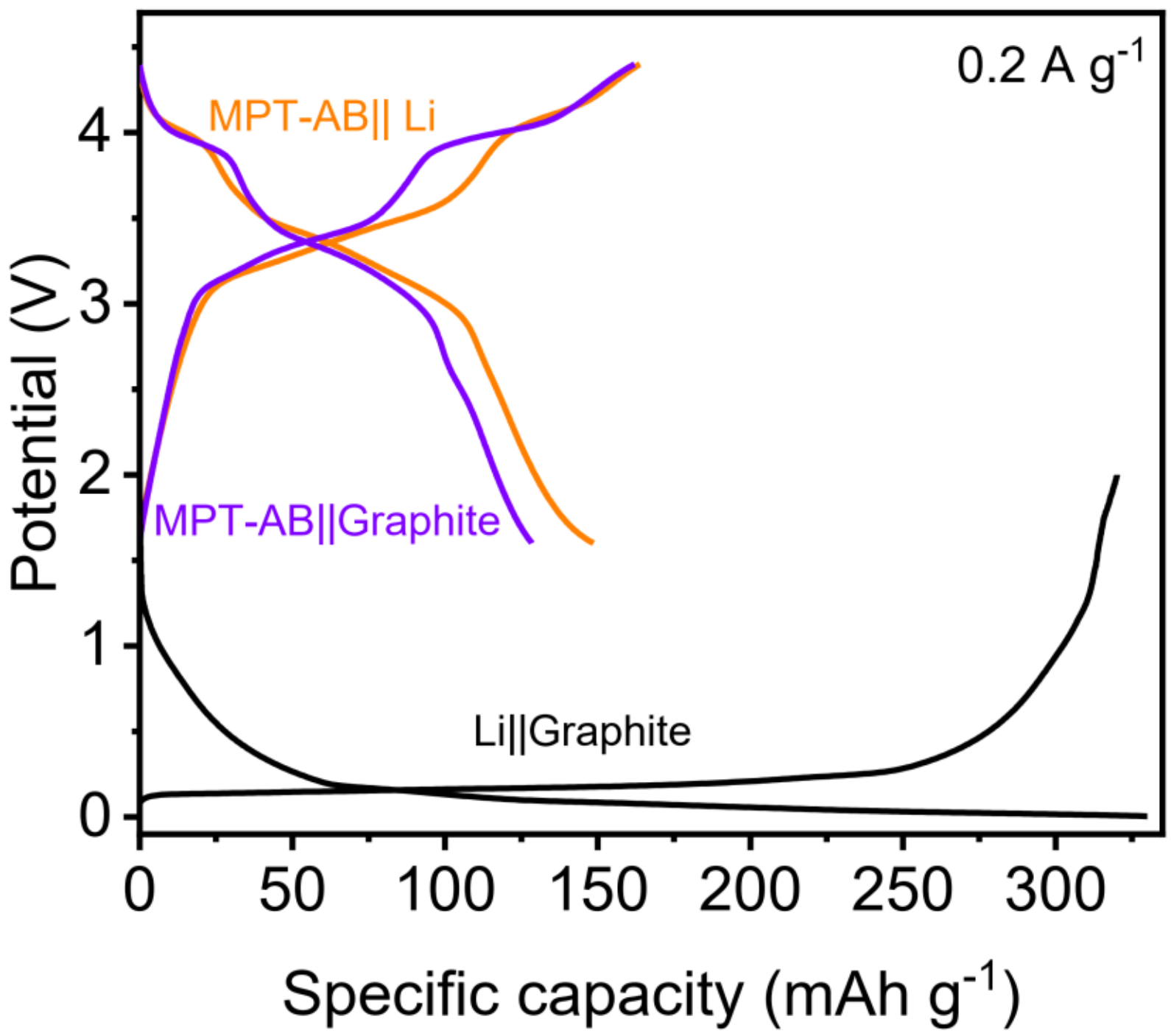

Figure S14. Charge–discharge curves of MPT-AB//graphite full cell, MPT-AB//Li half-cell, and graphite//Li half-cell.

Table S1. Summary of half-cell LOB performances for MPT-AC, MPT-AB and MPT-AP.

Materials	Theor. e numbers	C_{theor} (mAh g^{-1})	Rate performance: Capacity (mAh g^{-1}) Current density	Cycle Stability: Capacity (mAh g^{-1}) / retention / cycles / current density (A g^{-1})
**MPT-AC**	2	141	118, 100, 96, 89, 81<br>0.2, 0.5, 1.0, 2.0, 5.0	125/100%/500/0.2<br>83/80%/2000/5.0<br>72/70%/5000/5.0
**MPT-AB**	2	144	150, 127, 121, 114, 103<br>0.2, 0.5, 1.0, 2.0, 5.0	152/100%/500/0.2<br>99/90%/2000/5.0<br>79/72%/5000/5.0
**MPT-AP**	2	143	179, 148, 136, 128, 115<br>0.2, 0.5, 1.0, 2.0, 5.0	138/77%/500/0.2<br>31/27%/5000/5.0

Table S2. Electrochemical comparison of current work and other reported phenothiazine-based organic cathodes in the literature for LOBs.

**Materials** (reference)	**Electrode Composition**	C_{thero} (mAh g^{-1})	**Specific capacity** (mAh g^{-1}) **(Current)**	**Cycle Stability** Retention /Cycles / Current	**Redox Window** (V)
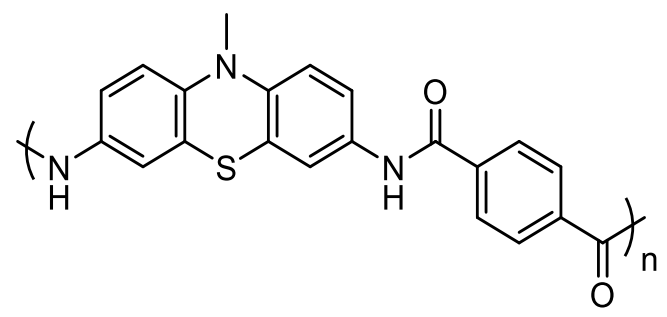<br>**MPT-AB**<br>**This work**	active materials: Ketjen Black: PTFE = 6:3:1	144	150 (0.2 A g^{-1})<br>127 (0.5 A g^{-1})<br>121 (1.0 A g^{-1})<br>114 (2.0 A g^{-1})<br>103 (5 A g^{-1})	100%/500/0.2 A g^{-1}<br>72%/5000/5.0 A g^{-1}	1.6-4.4V
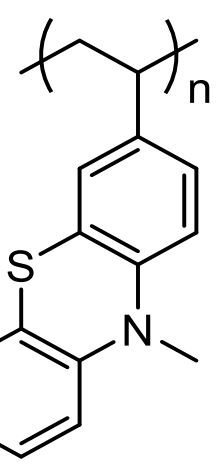<br>**PVMPT**<br>*Energy Environ. Sci.*, **2017**, *10*, 2334-2341.	active materials: Super P: PVDF = 5: 4.7: 0.3	112	56 (1 C)<br>50 (10 C)<br>26 (100 C)<br><br>1 C = 112 mAh g^{-1}	100%/1000/1 C<br>93.5%/10000/10 C	3-4 V
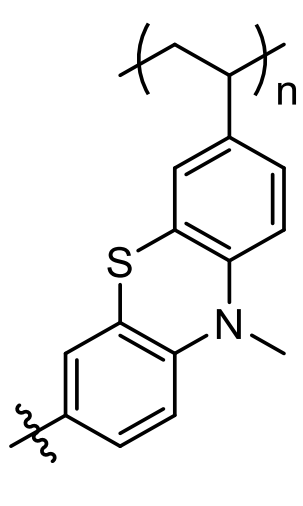<br>**X-PVMPT**	active materials: Super P: PVDF = 5: 4: 1	112	107 (1 C)<br>85 (10 C)<br>70 (20 C)<br><br>1 C = 112 mAh g^{-1}	95%/1000/1 C<br>95%/1000/10 C	3-4 V

Adv. Energy Mater. **2018**, *8*, 1802151.					
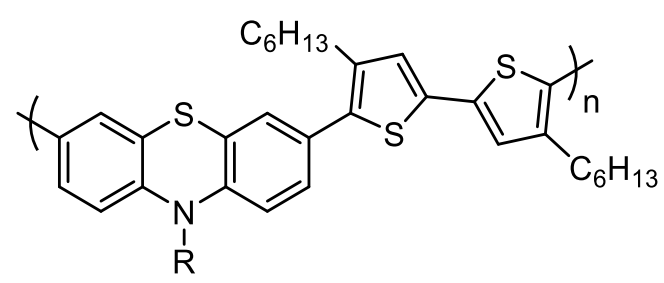<br>**P1a**<br>*Adv. Funct. Mater.* **2019**, *29*, 1906436.	active materials: Super P: PVDF = 6: 3.5: 0.5	36.4	34.7 (1 C)<br>32.7 (10 C)<br>1 C = 36 mAh g^{-1}	100%/100/1 C<br>100%/2200/10 C<br>97.5%/30000/100 C	3.2-3.9V
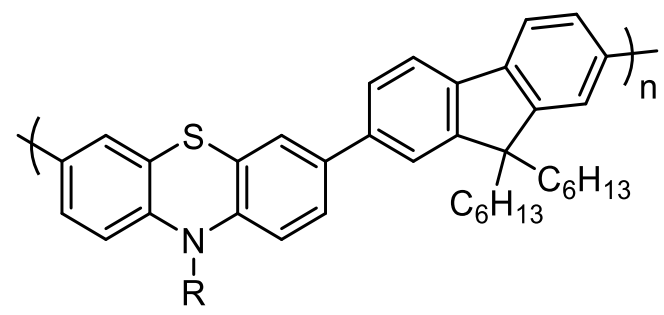<br>**P2**<br>*Adv. Funct. Mater.* **2019**, *29*, 1906436.	active materials: Super P: PVDF = 6: 3.5: 0.5	36.4	33.4 (1 C)<br>30.9 (10 C)<br>1 C = 36 mAh g^{-1}	98%/100/1 C<br>72%/2200/10 C	3.2-3.9V
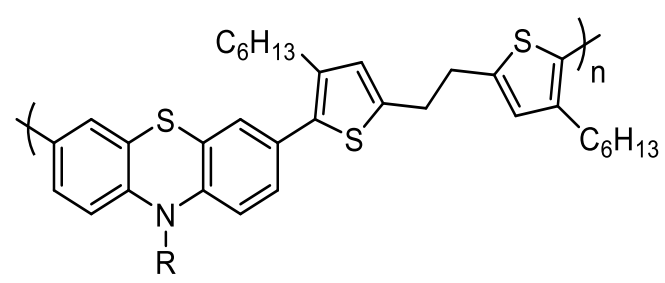<br>**P3**<br>*Adv. Funct. Mater.* **2019**, *29*, 1906436.	active materials: Super P: PVDF = 6: 3.5: 0.5	35.1	31.0 (1 C)<br>1 C = 35 mAh g^{-1}	100%/100/1 C	3.2-3.9V
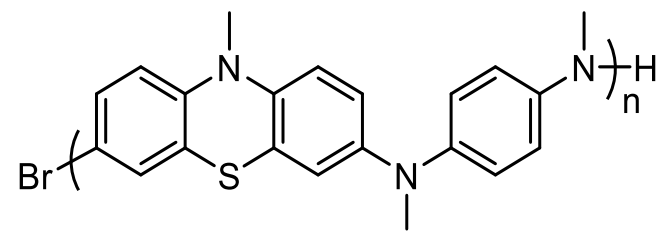<br>**PT-DMP**<br>*ACS Appl. Energy Mater.* **2018**, *1*, 3560-3564.	active materials: Super P: PVDF = 7: 2: 1	156	128 (1 C)<br>1 C = 156 mAh g^{-1}	64%/50/1 C	2.8-4.3V
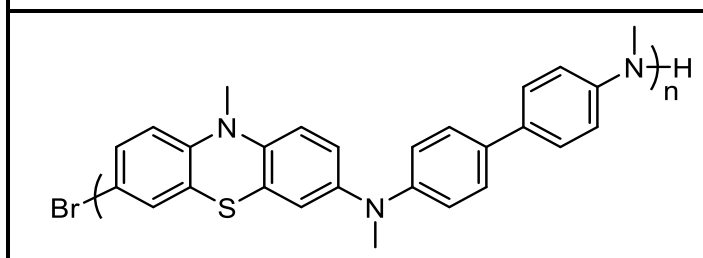<br>**PT-BZ**<br>*ACS Appl. Energy Mater.* **2018**, *1*, 3560-3564.	active materials: Super P: PVDF = 7: 2: 1	127	97 (1 C)<br>1 C = 156 mAh g^{-1}	66%/50/1 C	2.8-4.3V
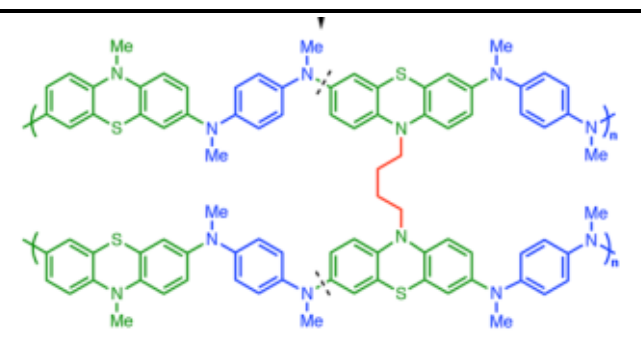<br>*ACS Appl. Energy Mater.* **2018**, *1*, 3560-3564.	active materials: Super P: PVDF = 7: 2: 1	155	150 (5 C)<br>1 C = 155 mAh g^{-1}	74%/50/5 C	2.8-4.3V
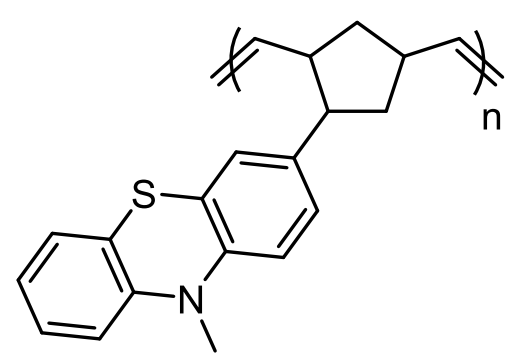<br>**PNMPT** (and **X-PNMPT**)<br>*ChemSusChem* **2020**, *13*, 2232-2238.	active materials: Super P: PVDF = 5: 4.5: 0.5	88	69 (1 C)<br>1 C = 88 mAh g^{-1}	97%/300/1 C<br>81%/1000/1 C	3.3-3.9V

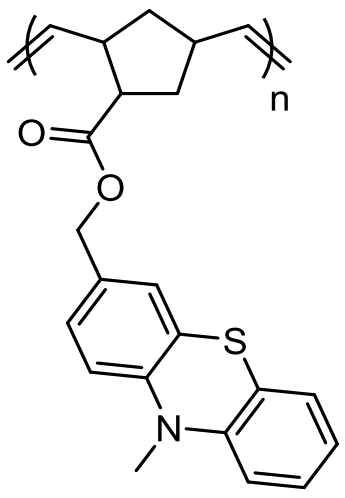<br>**P1**<br>*ChemSusChem* **2020**, *13*, 2232-2238.	active materials: Super P: PVDF = 5: 4.5: 0.5	74	62 (1 C)<br><br>1 C = 74 mAh g^{-1}	45%/300/1 C	3.3-3.9V
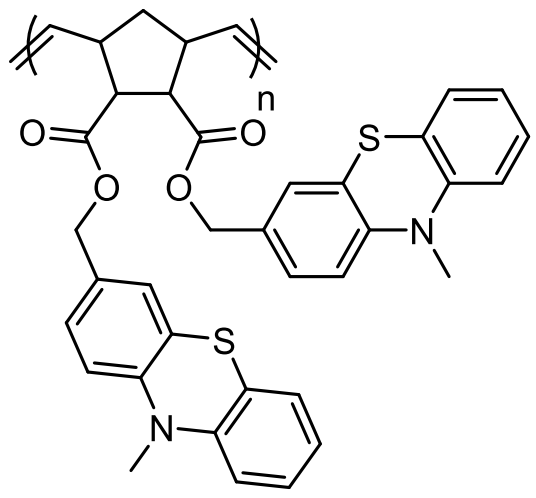<br>**P2**<br>*ChemSusChem* **2020**, *13*, 2232-2238.	active materials: Super P: PVDF = 5: 4.5: 0.5	86	73 (1 C)<br><br>1 C = 86 mAh g^{-1}	45%/300/1 C	3.3-3.9V
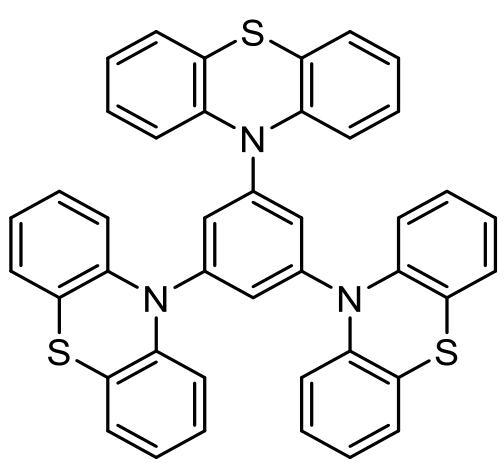<br>**3PTZ**<br>*Energy Environ. Sci.* **2020**, *13*, 4142-4156.	active materials: Super P: PVDF = 4: 4: 2	120	31 (1 C)<br>9 (20 C)<br><br>1 C = 120 mAh g^{-1}	66%/100/1 C	3-4V
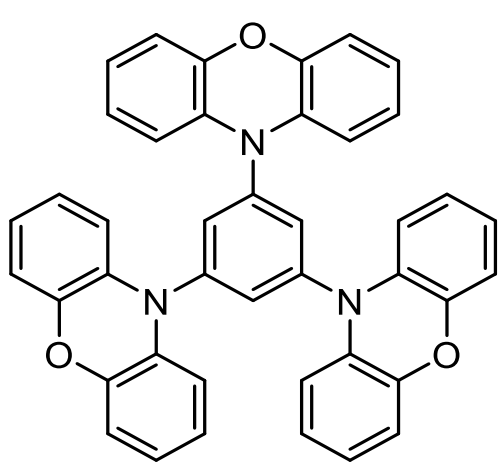<br>**3PXZ**<br>*Energy Environ. Sci.* **2020**, *13*, 4142-4156.	active materials: Super P: PVDF = 4: 4: 2	129	99 (1 C)<br>76 (20 C)<br><br>1 C = 120 mAh g^{-1}	65%/100/1 C	3-4V
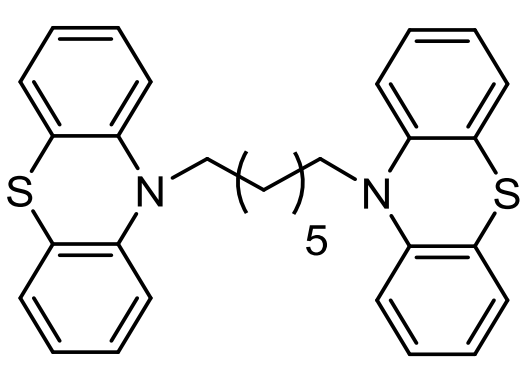<br>**2PTZ-C_7H_{14}**<br>*Adv. Mater.* **2024**, *36*, 2312486.	active materials: Super P: PVDF = 4: 4: 2	104	98 (1 C)<br>64 (10 C)	60%/1000/1 C	3-4V

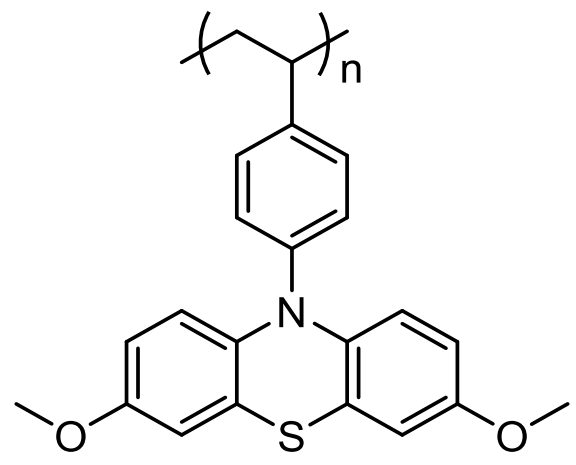 **P-PhPTZOMe** *J. Power Sources* **2024**, *612*, 234779.	active materials: Super P: PVDF = 9: 1: 1	148	109 (1 C)<br>101 (10 C)<br>94 (20 C)<br><br>1 C = 148 mAh g^{-1}	81%/800/1 C	2.8-4.1V

References

[S1] Frisch, M. J.; Trucks, G. W.; Schlegel, H. B.; Scuseria, G. E.; Robb, M. A.; Cheeseman, J. R.; Scalmani, G.; Barone, V.; Petersson, G. A.; Nakatsuji, H.; Li, X.; Caricato, M.; Marenich, A. V.; Bloino, J.; Janesko, B. G.; Gomperts, R.; Mennucci, B.; Hratchian, H. P.; Ortiz, J. V.; Izmaylov, A. F.; Sonnenberg, J. L.; Williams-Young, D.; Ding, F.; Lipparini, F.; Egidi, F.; Goings, J.; Peng, B.; Petrone, A.; Henderson, T.; Ranasinghe, D.; Zakrzewski, V. G.; Gao, J.; Rega, N.; Zheng, G.; Liang, W.; Hada, M.; Ehara, M.; Toyota, K.; Fukuda, R.; Hasegawa, J.; Ishida, M.; Nakajima, T.; Honda, Y.; Kitao, O.; Nakai, H.; Vreven, T.; Throssell, K.; Montgomery, J. A., Jr.; Peralta, J. E.; Ogliaro, F.; Bearpark, M. J.; Heyd, J. J.; Brothers, E. N.; Kudin, K. N.; Staroverov, V. N.; Keith, T. A.; Kobayashi, R.; Normand, J.; Raghavachari, K.; Rendell, A. P.; Burant, J. C.; Iyengar, S. S.; Tomasi, J.; Cossi, M.; Millam, J. M.; Klene, M.; Adamo, C.; Cammi, R.; Ochterski, J. W.; Martin, R. L.; Morokuma, K.; Farkas, O.; Foresman, J. B.; Fox, D. J., Gaussian, Inc., Wallingford, CT, USA, **2016**.

[S2] Lindström, H.; Södergren, S.; Solbrand, A.; Rensmo, H.; Hjelm, J.; Hagfeldt, A.; Lindquist, S.-E. Li^+ Ion Insertion in TiO_2 (Anatase). 2. Voltammetry on Nanoporous Films. *J. Phys. Chem. B* **1997**, *101*, 7717-7722.

[S3] Liu, T. C.; Pell, W. G.; Conway, B. E.; Roberson, S. L. Behavior of Molybdenum Nitrides as Materials for Electrochemical Capacitors: Comparison with Ruthenium Oxide. *J. Electrochem. Soc.* **1998**, *145*, 1882-1888.

[S4] Li, H.; Chen, L.; Xing, F.; Miao, H.; Zeng, J.; Zhang, S.; He, X. Cross-linking enhances the performance of four-electron carbonylpyridinium based polymers for lithium organic batteries. *Chem. Sci.* **2024**, *15*, 14399-14405.
